# Self-Healing, Electroconductive Hydrogels for Wound Healing Applications

**DOI:** 10.3390/gels11080619

**Published:** 2025-08-08

**Authors:** Duarte Almeida, Diogo Dias, Frederico Castelo Ferreira, Teresa Esteves

**Affiliations:** 1 Institute for Bioengineering and Biosciences, Department of Bioengineering, Instituto Superior Técnico, Universidade de Lisboa, Av. Rovisco Pais, 1049-001 Lisbon, Portugal; duarte.almeida@tecnico.ulisboa.pt (D.A.); diogodias211@gmail.com (D.D.); frederico.ferreira@tecnico.ulisboa.pt (F.C.F.); 2 Laboratory i4HB, Institute for Health and Bioeconomy at Instituto Superior Técnico, Universidade de Lisboa, 1049-001 Lisbon, Portugal

**Keywords:** wound healing, hydrogel, electrical stimulation, self-healing capability, cell proliferation

## Abstract

Electroconductive, self-healing hydrogels have surfaced as a versatile tool for advanced wound care applications, since they combine classic hydrogels’ moist and biomimetic environment with the dynamic electrical responsiveness that can function as an accelerator of tissue repair processes. Recent advances report the automatic restoration of materials after mechanical disruption through various mechanisms, such as ionic or covalent bonds and supramolecular interactions. This property is crucial for biomaterials, as they are often applied in skin regions with high motility and, therefore, a high risk of breakage. By integrating within these networks compounds that are electrically active—polymers such as PEDOT:PSS or polypyrrole, or 2D nanomaterials such as graphene—it is possible to confer responsiveness to these hydrogels, which can lead to increases in fibroblast proliferation, antimicrobial properties, and angiogenesis. Furthermore, these biomaterials must have skin-mimicking mechanical properties and can also be loaded with drugs to improve their healing properties even further. This review synthesizes the chemistry behind the self-healing and electroconductive properties of these materials and expands on the available literature on this field and their biological outcomes, while also providing a look into the future of these promising materials, aiming at their integration in standard wound care strategies.

## 1. Introduction

Skin is the body’s largest organ [1], with functions such as thermo- and immune regulation, stimuli sensitivity, maintenance of water levels [2], and protection from pathogens. Its continuous exposure to the external environment renders it highly susceptible to injury. Although in normal circumstances skin has several repair mechanisms that allow it to heal rapidly and efficiently [3], in severe wounds, this healing frequently comes without the proper regeneration of skin appendages—hair follicles, sweat and sebaceous glands—with scar tissue replacing the original skin [4]. Normal skin repair follows a regulated sequence of hemostasis, inflammation, proliferation, and remodeling [2,3]. Nevertheless, factors such as excessive inflammatory signaling [5], diabetes, or the inability to move can hinder this cascade and lead to the formation of chronic wounds [6]. Modern wound dressings are, hence, designed not only to cover and protect the lesioned area from external infections, but also to guide cell reorganization and their integration into host tissues. To have these effects, a dressing must be (1) biocompatible and non-irritant;, (2) maintain moisture of the area while, at the same time, absorb exudate; (3) have adequate mechanical properties to sustain its shape without losing function; and (4) have features that promote cell adhesion, proliferation and, if that is the case, differentiation [7].

Several options have been investigated—from semi-permeable films and foams, to nanofibers and hydrocolloids [8]—with hydrogels taking a standout position [9]. Hydrogels are 3D polymeric structures of natural or synthetic origin, characterized by a high water content [10] and especially conducive to tissue regeneration [11].

It is well known that the growth and function of cells and tissues can be affected by electrical signals and that an important contributor to skin wound healing are the endogenous electrical fields that are generated upon this type of injury [2,3]. Nevertheless, traditional hydrogels are usually not conductive and, as such, there is a need to introduce conductive components into these 3D matrices to create electroconductive hydrogels [12,13]. These components can range from π-conjugated polymers, such as polypyrrole (PPy) and polyaniline (PANI), to carbon-based nanostructures, such as graphene and carbon nanotubes (CNTs) [12].

Another topic that is not frequently covered is the need for new dressings that can be used in wounds in body parts subjected to stretching and/or bending [14], such as joints like the elbow, knee, fingers, or wrist. Conventional dressings, such as bandages, cause discomfort and inconvenience to the patients and due to their unstable connection to the wound site, they also have decreased reliability [15]. Self-healing hydrogels, with their native ability to regenerate their initial structure upon damage, can increase the service time of the dressing, reducing costs and patient inconvenience, while also providing the ideal environment for the wound to heal [16,17].

**Figure 1:**
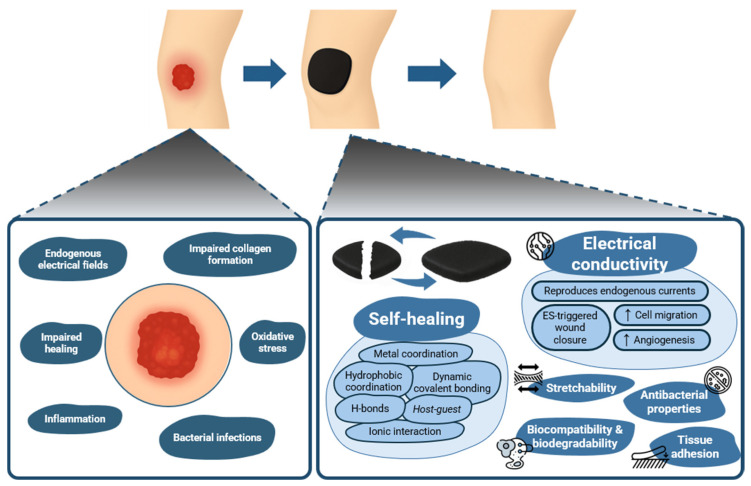
Self-healing, electroconductive hydrogels for wound healing: from injury to repair.

As such, there is an evident advantage in combining electroconductivity with self-healing properties for the development of hydrogels for wound healing. In spite of this, as far as we are aware, the literature lacks an overview of hydrogels combining both these properties for wound healing applications. Hence, this review will first provide an overview of the wound healing process, followed by clarification on the self-healing properties of hydrogels as wound dressings and how this process occurs, as well as different strategies and elements commonly used to impart electroconductivity into these hydrogels. With this work, we aim to highlight how the different bonding chemistries and conductive components compare in terms of mechanical properties, self-healing efficiency, and ability to provide suitable currents that can clinically promote wound healing. Finally, the current state-of-the-art will be laid out, and a future outlook into these materials, their promise, and their pathway to clinical use will be provided, while also showcasing what separates the investigated constructs from commercial wound dressings. An overview of this concept is illustrated in Figure 1, summarizing the wound environment, the hydrogel’s properties, and the healing outcomes.

## 2. Wound Healing Biology

**Figure 2:**
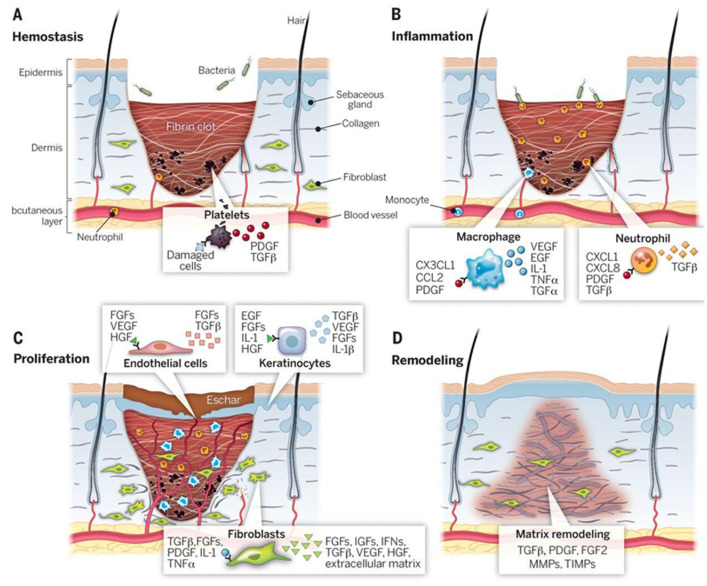
Four stages in wound healing: (**A**) hemostasis, (**B**) inflammation, (**C**) proliferation, and (**D**) remodeling. Reprinted with permission from ref. [9]. Copyright 2018 Elsevier.

To better understand the rationale behind the design of these advanced hydrogels, it is important to first revisit the biological stages of wound healing. As stated earlier, skin tissue repair occurs through a dynamic set of processes—hemostasis, inflammation, proliferation, and remodeling (Figure 2) [2,3]—which are affected by the electric field created at the wound sites [18]. In this section, the main processes of each wound healing stage will be explained, and the role of electric fields in each of them will be highlighted.

The first stage is hemostasis (Figure 2A). The goal of this first cascade of processes is to mitigate blood loss: the body reacts by promoting vasoconstriction in the injured area, and the platelets that circulate in the blood become activated, adhering to each other and to the newly exposed ECM. This initiates the process of fibrin crosslinking, which ultimately results in the binding of the aggregated platelet plug, forming the thrombus that stops the bleeding [19] and protects the wound from microorganisms [3]. Although the pool of evidence showing the effects of electric fields in this stage is small, studies have shown their potential to activate platelet surface procoagulant markers, promoting hemostasis [18].

Secondly, the inflammatory phase takes place (Figure 2B). Monocytes and neutrophils are able to reach injury sites, where they stimulate the production of molecules that increase endothelial cell permeability, thereby facilitating the movement of inflammatory cells at the site. Neutrophils flood the site with reactive oxygen species (ROS) and antimicrobial peptides [19], and they phagocyte external agents in a way that is dependent on the strength of the endogenous electric fields formed [18]. These electric fields are generated by the disruption of the transepithelial potential that occurs after damage, typically range from 40 to 200 mV·mm^−1^, and result from the directed transfer of ions by epithelial cells [20]. The generated current plays an important role in wound healing by guiding the directional migration of keratinocytes and fibroblasts (a phenomenon known as galvanotaxis), modulating inflammatory responses and promoting angiogenesis [20]. Subsequently, monocyte-derived macrophages are chemotactically attracted to the wound area and eliminate apoptotic neutrophils—essential for the wound not to evolve to a chronic condition. These cells, then, change their phenotype from pro- to anti-inflammatory, which is key for the release of tissue reconstruction factors such as arginase, interleukin (IL)-10, and angiogenesis factors [3], as well as electric field-mediated release of tumor necrosis factor alpha (TNF-α), neurotrophin 3 (NT3), IL-1, and IL-23 [18].

In the following days, the proliferation phase (Figure 2C) dominates the wound healing response. Keratinocytes located at the wound’s edge, partly activated by injury-induced electric fields, undergo a partial epithelial-to-mesenchymal transition (EMT) and move laterally to promote the closure of the epidermis [18,21,22]. In the meantime, fibroblasts replace the fibrin plug with granulation tissue, and hypoxia-induced vascular endothelial growth factor (VEGF) release promotes angiogenesis in the injured site. While these processes take place, macrophages regulate ECM deposition and the stability of the newly formed capillary networks. Peripheral nerve repair also takes place, driven by the exceptional plasticity of Schwann cells that can redifferentiate after damage and promote the rebuilding of axons, which is also supported by the recently organized neovasculature [19].

Finally, in the remodeling phase (Figure 2D), which can last for months or even years, collagen III, which first populated the wound region, is gradually replaced by collagen I, which is stronger [19]. The resulting scar, however, does not usually recover more than 80% of the tensile strength of uninjured skin and often also lacks the native skin appendages [3]. Electric fields can accelerate wound remodeling and also decrease the formation of scar tissue through the inhibition of the TGF-β/SMAD signaling pathway [18].

Understanding the wound healing process is key to designing proper wound dressings. An ideal wound dressing needs to interact—but not disrupt—the initial clot, regulate excessive inflammation, remain mechanically resilient through the entire process, and provide bioactive cues that can accelerate keratinocyte migration and angiogenesis.

## 3. Self-Healing Hydrogels: Mechanisms

**Figure 3:**
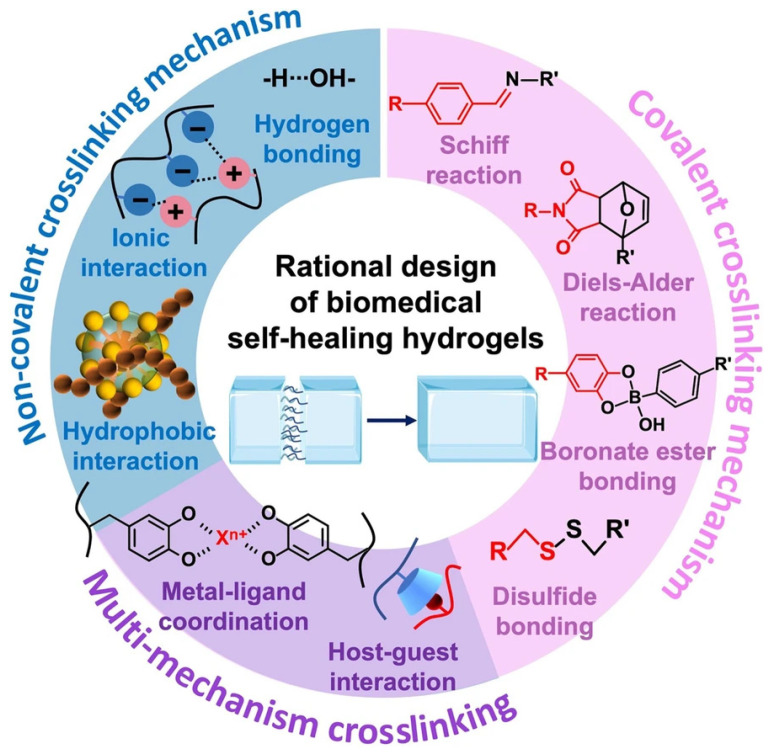
Self-healing chemistries and mechanisms for self-healing hydrogels: dynamic covalent bonds, non-covalent interactions, and multi-mechanism interactions. Reprinted with permission from ref. [34].

Self-healing hydrogels are materials that are able to regenerate after mechanical damage. This behavior stems from reversible covalent and non-covalent bonds that allow the polymer network to reorganize and restore its integrity. The major self-healing mechanisms used in hydrogel design are summarized in Figure 3. Given that these materials can regain their native organization, they can retain their mechanical performance for a longer period than conventional gels, even when subjected to repeated stresses. Often, self-healing hydrogels are formulated so that they are injectable, given that they recover their properties after being injected, which can allow painless dressing placement that conforms to complex wound geometries while keeping the wound site undisturbed. Therefore, due to their extended service life, reduced needs for replacement, and less risk of fatigue-related failure, these materials improve safety and cost-effectiveness, rendering them very attractive for several biomedical areas—from sustained drug release to next-generation wound dressings [16,23].

Self-healing can be triggered autonomously, or it can be prompted by an external stimulus. The former occurs when the energy required to promote the re-formation of bonds is low enough; the latter, on the other hand, happens when the energy must be supplied by an external agent, such as heat, light, pH, catalysts, or other types of physical stimuli such as ultrasounds (US) and electromagnetic fields [24]. Autonomously self-healing hydrogels are advantageous because they often are quick to regenerate, whereas stimuli-responsive self-healing imparts the advantages of increased control over the process. Furthermore, stimuli-induced self-healing has the ability to heal cracks that have been exposed to air for a long time, which is not usually achievable in autonomously self-healing hydrogels [24].

Light has great potential as a targeted stimulus for self-healing [25,26,27,28], since photons can supply energy only to the required areas, with high spatial and temporal control [29]. Hydrogels can harness ultraviolet (UV) radiation to cleave and re-form bonds between opposite ends of the broken material, or they can contain light-absorbing components that, upon irradiation, release heat that decreases the viscosity of the materials and increases the diffusion of chains across the broken ends. Although this strategy allows fast, efficient, and highly tunable healing, it is limited by its penetration ability in thicker and more opaque hydrogels [24]. pH and catalyst agents can also impact the self-healing properties of the hydrogels by increasing or decreasing the availability of functional groups, which promotes bond formation at the damaged interfaces [30]. This healing can be tailored, as well, by adjusting the catalyst dose or pH value and the contact time with the hydrogel, despite also carrying its disadvantages, such as a potential slow self-healing efficiency and possible damage to the performance or morphology of the hydrogels [24].

Temperature is another stimulus used to trigger the self-healing of hydrogels. For instance, Zhang et al. [31] developed an injectable hydrogel responsive to dual stimuli (temperature and pH), which showed robust mechanical performance and self-healing ability at 60 °C and subsequent cooling. Deng et al. [32] reported a hydrogel capable of mechanical recovery under cyclic thermal stimulation, achieving healing efficiencies above 90% after mild heating. More details about these studies are provided in this manuscript. It should be noted that temperature is a significant factor in the reversibility of certain chemical reactions. For instance, Diels–Alder adducts undergo reversible formation in organisms requiring the use of thermal cycling for bond breakage and reformation that does permit efficient healing but must be designed to operate under physiologically safe temperatures for biomedical applications.

Finally, remotely stimulated self-healing has been gaining traction in the scientific community, namely, by the use of US, magnetic fields, or electricity. Since these stimuli are delivered without direct contact with the material, they allow self-healing in situ, which is highly advantageous for bulkier constructs or hydrogels placed in internal locations [17,24].

US causes hydrodynamic cavitation within hydrogels, which produces intense pressure and temperature gradients. This energy dissipates, originating free radicals that react with the polymeric chains and reconfigure the network of the hydrogel, healing the damage. This type of stimulus has a superior penetration degree while maintaining relatively high spatiotemporal resolution [33]. Magnetic field-triggered self-healing hydrogels usually encompass the introduction of superparamagnetic or ferromagnetic components in the hydrogel that, upon magnetic stimulation, increase the temperature in situ and, thus, increase polymer chain diffusivity that can repair the damaged area [31]. Electricity-responsive mechanisms resort to the electrothermal effect of materials like CNTs to trigger heat-responsive self-healing [24]. These approaches pave the way for wireless and localized repair of hydrogels, with several applications.

### 3.1. Dynamic Covalent Bonding

In this type of hydrogel, there are covalent bonds that have the possibility to break and reconnect, upon damage to their network, under mild conditions [23]. When the material is cut or broken, the same equilibrium that allows hydrogel gelation also allows its autonomic repair. Common dynamic covalent bonds used in self-healing hydrogels include imine bonds, disulfide bonds, and boric acid ester linkages [23].

The imine bond—also referred to as Schiff base bond—results from the condensation reaction between an amine (-NH_2_) and an aldehyde (-CHO) or ketone (-CO-) group (widely present in polymers like chitosan (CS), or collagen) forming a carbon–nitrogen double bond that is classified as a Schiff base (aliphatic or aromatic). For instance, injectable self-healing hydrogels were created by joining chondroitin sulfate multiple aldehydes and *N*,*O*-carboxymethyl chitosan (CMCS)/oxidized chondroitin sulfate, which showed antibacterial properties while maintaining the hemostatic condition of wounds [35]. This bond is the most popular due to its ease of occurrence under standard physical conditions, the wide range of sources for its functional groups, and its inherent self-healing characteristics within hydrogel systems, thus conferring extensive application potential [23,36,37], among which wound healing stands out [38,39,40,41].

This type of bond is also used in exploring the employment of green biological crosslinkers, obtained from natural resources, which emerge as an alternative to the potential toxic effects of synthetic crosslinking agents [42], ensuring higher safety for humans and the environment. This way, the oxidized form of a green biological crosslinker (konjac glucomannan), along with CS, followed a Schiff base interaction for the creation of a hydrogel, with high recovery after breaking, thereby revealing its self-healing capability [43]. Similarly, a natural wound dressing material was synthesized with CS and dialdehyde bacterial cellulose and presented injectability, good mechanical strength, drug delivery, antibacterial activity, and a dynamic self-healing ability due to the imine bond formation, making it a superior material for tissue engineering applications, particularly wound dressing [44]. Finally, a recent study by Wang et al. [45] introduced a biocompatible hydrogel wound dressing, synthesized through a green chemistry approach, designed specifically to meet the needs of acute and chronic wound care. The strategy employed sustainable biomaterials, soy protein, and vanillin (antimicrobial and antioxidant properties), to create a physical–reversible chemical dual-crosslinked hydrogel, which exhibited high mechanical strength, adhesion, and toughness. Schiff base reversible bonds allowed rapid self-healing (<10 s). The developed material showed strong hemostatic performance, antioxidant activity, and accelerated healing of diabetic wounds in an in vivo model [45].

Interestingly, a nanocomposite DNA-based hydrogel crosslinked with oxidized alginate (OA) via the formation of reversible imine linkages was reported [46]. The hydrogel formulations exhibited self-healing and shear-thinning properties due to the reversible nature of the covalent imine bonds formed between the aldehyde groups of OA and the amine groups present in the DNA nucleotides. Incorporation of charged silicate-based nanoparticles (NPs) increased the shear strength of the prepared hydrogels by establishing electrostatic interactions with the phosphate groups of the DNA network. These hydrogels were also able to perform sustained drug delivery. Therefore, DNA can be explored as a natural biopolymer to fabricate self-healing injectable hydrogels with sustained release properties for minimally invasive therapeutic approaches [46]. Due to their inappropriate amino groups, gelatin-based hydrogels based on Schiff base reactions are uncommon. In order to increase the amino group of gelatins, a simple method that involves creating a hydrogel with ethylene diamine has been researched. It enhanced the capacity for self-healing by crosslinking with dialdehyde carboxymethyl cellulose [47].

Acylhydrazone bonds, formed by reversible condensation between hydrazine or hydrazide and aldehyde/ketone, are very close relatives to imine bonds, being also spontaneously formed under physiological conditions [17]. These bonds can be formed as a result of hydrolysis or exchange reactions [17]. The acylhydrazone bonds formed by the reaction between the aldehyde group and the hydrazone bonds of oxidized dextran and acid dihydrazide, respectively, with the gold-standard antimicrobial agent chlorhexidine acetate, with prolonged release of basic fibroblast growth factor (bFGF), resulted in improved wound healing [48].

Other dynamic covalent bonds type used in self-healing hydrogels, although weaker than imine bonds, are disulfide bonds. In the presence of nucleophilic thiolates, the thiol/disulfide exchange process forms disulfide bonds in a neutral or alkaline environment [49]. The disulfide bonds experience an exchange reaction, where S-S neighboring bonds become disrupted and reformed through ionic intermediates [50]. These stable bonds are formed through thiol (-SH) oxidation and are sensitive to reduction by reducing agents, allowing the creation of redox-responsive sol–gel systems [32]. Such redox sensitivity is particularly advantageous for the wound healing process, as the glutathione-rich reducing environment of wounds favors disulfide exchange [16,17]. Additionally, this bond is pH- and light-responsive. Chen et al. [51] created a composite hydrogel with silver nitrate (AgNO_3_) and thiolated poly(ethylene glycol) (PEG) that has antibacterial properties and can withstand external mechanical resistance in diabetic wound repair. Ag^+^ improved the bactericidal action, while the disulfide bond of Ag-S improved the material’s capacity for self-healing, making it a viable material for diabetic wounds [51].

Diels–Alder reactions have also been implemented by researchers to yield self-healing hydrogels. This reaction consists of the [2+4]-cycloaddition of compounds with a conjugated system of double bonds (diene) with compounds having a double or triple bond (dienophilic), leading to the formation of a six-membered cycle [52]. This type of reaction occurs in the absence of a catalyst or coupling reagent and is thermoreversible, breaking at high temperatures (>100 °C), and regenerating after temperature decrease [17]. Thermosensitive hydrogels based on this reaction paved the way for further research on their use in tissue engineering [53] and drug delivery [54].

Extensive research has confirmed the employment of boronic acids and their derivatives, such as phenylboronic acid (PBA), in the synthesis of self-healing hydrogels [23]. The reaction behind it is boronate ester complexation, which is a reversible boronate ester bond that is formed by the condensation reaction between boronic acids and 1,2- or 1,3-diols at the pH near or higher than the pKa of the boronic acids [17,23]. Diols such as glycopolymers [55,56], poly(vinyl alcohol) (PVA) [57], catechol-containing compounds [58,59], and polysaccharides [60] have been reported for boronic ester-based hydrogels. Because the boronic acid–diol complexes undergo reversible hydrolysis/reformation, hydrogels created using these crosslinking techniques are transient [61].

Despite being highly pH-sensitive, these bonds can be used to develop self-healing hydrogels that are tissue and cell-compatible [32,37], with promising results towards wound healing [62]. For instance, a PVA-borax hydrogel reinforced with natural antibiotic resveratrol-grafted cellulose nanofibrils employs reversible borate ester bonds (combined with hydrogen bonding), achieving > 90% self-healing efficiency, pH-responsive drug release, antioxidant function, and enhanced healing in bacteria-infected wound models [63].

Each type of covalent bonding is associated with a specific healing time and ability to recover mechanical properties. Imine bond-mediated self-healing has been reported to occur in as little as 1 min after the ends of the cut hydrogel are put in contact [64], and they also show ability to immediately recover their storage modulus after being subjected to high strains [38,65,66]. Self-healing induced by boronate ester complexation has been shown to take longer than the previous one, with Ali et al. reporting that full healing occurs in 20 min, and first contact occurs after 5 min [67]. Nevertheless, it is worth pointing out that comparisons among different types of self-healing mechanisms, in terms of their time to self-healing and recovery of mechanical properties, are hard to achieve. This occurs because hydrogels often combine several of these mechanisms to achieve a higher self-healing efficiency, and, as such, it is difficult to establish which mechanism is mainly driving self-healing.

By selecting and combining these chemistries, it is possible to tailor hydrogels to keep their required purposes for a wide set of demanding applications.

### 3.2. Ionic Interaction

Ionic bonds can also be exploited for the construction of self-healing hydrogels by reversible electrostatic interaction between oppositely charged moieties [23,24,32].

An example is alginate hydrogels formed from negatively charged alginate pre-polymers, which are crosslinked into a hydrogel via divalent ions, usually calcium. This is a classic ionic interaction-based self-healing hydrogel and is suitable for wound dressings [17,68,69]. Less commonly, one can utilize electrostatic interactions of charged nanomaterials—such as poly(3,4-ethylenedioxythiophene):polystyrene sulfonate (PEDOT:PSS), for instance—with the hydrogel backbone, because it may introduce a variety of additional features to the system due to the multifunctional nature of the nanomaterials [17].

These ionic interaction-based self-healing hydrogels possess much higher conductive ionic, electronic, or mixed conductivity. Nonetheless, their long-term stability and mechanical integrity may still be inferior to that of covalently crosslinked materials. Therefore, scientists frequently integrate this type of interaction with other dynamic chemistries to share mechanical loads and enhance performance [68].

### 3.3. Hydrogen Bonds

Hydrogen bonding (H-bonding) is a type of intermolecular force that occurs when a hydrogen atom covalently linked to a highly electronegative atom establishes an electrostatic link with another electronegative atom (oxygen, nitrogen, or fluoride). Although H-bonds are weaker than covalent and ionic bonds, they still contribute considerably to the mechanical properties of hydrogels when present within the hydrogel matrix [68]. Furthermore, H-bond formation and dissociation happen quickly, being one of the main causes for the rapid healing time observed in self-healing hydrogels based on these interactions. A prolonged exposure to low pH values has been reported to promote intramolecular H-bonding, which decreases the availability to form this type of interaction with molecules across the damaged surface. In this way, exposure to increased pH values can reverse this effect and promote hydrogel self-healing [70].

The most well-known example consists of PVA, and polyacrylamide (PAM), with the H-bonds being mediated by either hydroxyl or amide groups [17,23,71,72]. This interaction is also critical for the development of self-healing hydrogels of cationic guar gum (GG), which contains numerous hydroxyl groups and branches for effective intermolecular hydrogen bonding. An injectable self-healing conductive slime based on cationic GG has been reported, providing a means for wound healing and tissue regeneration within 1 min [73].

### 3.4. Hydrophobic Interaction

These interactions occur when hydrophobic groups aggregate to avoid contact with water. The self-healing found in hydrogels containing hydrophobic moieties is due to reversible interactions between such moieties. For instance, in a hydrogel composed of a hydrophobically modified polymer and also hydrophobic NPs, the interactions among them promoted the self-healing process of the hydrogel [74]. Other studies demonstrated that mechanical properties could also be improved by combining H-bonds with a hydrophobic cage structure. After constructing large hydrophobic groups copolymerized with a hydrophile like acrylamide, the self-healing hydrogel exhibited higher toughness [75,76].

When surfactants are involved, micelles—spheres of hydrophobic groups surrounded by amphipathic molecules—work as crosslinking backbones and enable better self-healing [77,78]. This self-healing performance is due to the dynamic break and reformation of the micelles.

### 3.5. Metal Coordination Interaction

When metal cations act as electron acceptors and ligands supply the electrons, reversible metal coordination interactions occur. Metal ions such as Fe^3+^, Zn^2+^, and Ca^2+^ can form coordination complexes with organic ligands (-COOH groups present in polymers like alginate, oxidized cellulose nanofibers, and poly(acrylic acid) (PAA)), creating dynamic crosslinks that can break and reform under stress [68].

One of the most reported strategies of metal coordination for self-healing hydrogels is based on the adhesiveness of the mussel [68]. Fe^3+^-catechol coordination (chelation) provides both strong adhesion and self-healing capabilities [79]. For instance, a pH-sensitive Fe^3+^-catechol coordinated bond was developed in a dual-dynamic-bond hydrogel. It is an ideal dressing for infected skin wounds, especially when combined with quaternized CS (QCS), which has an antibacterial effect [80]. Fe^3+^ has also been used to coordinate with carboxylate groups in the synthesis of a salecan polysaccharide-based hydrogel in the work of Deng et al. [81]. This hydrogel had injectable, shear-thinning, and skin-adhesive properties, with high porosity and water content (90%). The hydrogel was also cut, with its pieces being able to heal in under 30 min without external intervention. Fe^3+^ could be photoreduced to Fe^2+^ by 365 nm irradiation, which triggered gel-to-sol conversion and allowed easier removal of the dressing from wounds. Furthermore, it showed antibacterial activity, while retaining fibroblasts viability, and it improved wound closure rate (94% within 12 days) in a rat full-thickness wound model.

Zinc coordination offers additional therapeutic benefits due to this element’s essential role in wound care and its antimicrobial properties. Taking advantage of this, Li et al. [82] used Zn^2+^ and Ag^+^ ions to dynamically crosslink a hydrogel based on thiolated hyaluronic acid and on a vasculogenic peptide (K_2_(SL)_6_K_2_-SH), naming it HA-S-Ag/Zn/KS. This material showed a porosity of around 67%, self-healed in 10 min after being cut, was shear-thinning, and was able to swell to 3400% its dry mass. Different components of HA-S-Ag/Zn/KS played different roles in the system: Ag^+^ was responsible for the bactericidal activity of the hydrogel—99% against *S. aureus* and *E. coli*—Zn^2+^ drove macrophage polarization to an anti-inflammatory phenotype, and the vasculogenic peptide sped endothelial cell migration and tube formation. When applied to infected full-thickness wounds in rat, the hydrogel promoted bacterial death, stimulated angiogenesis and collagen deposition, and closed wound almost completely in 14 days.

Besides these, many other pairings have been shown effective—Ni^2+^-pyridine [83], Ni^2+^-histidine [84], Au-thiolate [85], Ag^+^-biphosphonate [86], Zn^2+^-histidine [87], among others [88].

In summary, metal coordination-crosslinked hydrogels combine rapid autonomous self-healing with mechanical performance, with highly tunable properties by simply changing the metal or ligand used.

### 3.6. Host–Guest Interaction

In host–guest crosslinked hydrogels, there is typically a complex formed between a macrocyclic host and a guest molecule with complementary shape and physical/chemical properties. The host has an internal cavity with a certain size and polarity that is occupied by the guest molecule, by a combination of hydrophobic attraction (the most relevant), H-bonding, ionic contacts, and π–π stacking. When the hydrogel is damaged, the crosslinks at the interface are destroyed, but they can re-form once the two ends are put in contact again [23].

Research has been conducted on several potential hosts, with cyclodextrin (CD) dominating. CD is a molecule that consists of a lipophilic inner cavity and a hydrophilic outer surface that accepts a wide variety of hydrophobic guest molecules [17]. For instance, a self-healing conductive hydrogel incorporating β-CD as the host molecule, N-isopropylacrylamide (NIPAM) as the guest molecule, multiwalled CNTs (MWCNTs) as the physical crosslinker and conducting substrate, and nanostructured PPy as the highly conductive component, was developed [89]. This hydrogel exhibited high conductivity, flexibility, stable and elastic mechanical properties, excellent self-healing properties, and stimuli responsiveness.

These hydrogels can be prepared by grafting the host molecule onto one section of the polymer chains and the guest molecule onto a different region, or by introducing them into two different chains. Another way is by using supramolecular monomers with a pre-organized host–guest pair in combination with regular monomers or crosslinkers to generate self-healable networks. These materials can, thus, self-heal within hours at room temperature (RT), maintaining their adhesion properties nearly at the level of the pre-damage hydrogel [17,23].

The high binding selectivity, stimulus responsiveness, and ability to recognize specific molecules make host–guest-crosslinked hydrogels attractive for properties that require self-healing. Nevertheless, their mechanical strength is still not as high as in covalently linked materials.

Although each of these dynamic bonding mechanisms can impart hydrogels with autonomous self-healing capacity, it is increasingly common to combine more than one type of interaction [65,90,91,92,93,94,95] (e.g., imine bonds with H-bonding, boronic esters with coordination chemistry) to achieve improved healing efficiency, mechanical strength, or responsiveness in complex wound environments.

Despite recent advances, the literature comparing different self-healing chemistries alongside each other under identical conditions is still limited, which makes it difficult to draw meaningful structure–function relationships.

## 4. Designing Electroconductive Hydrogels for Wound Healing

**Figure 4:**
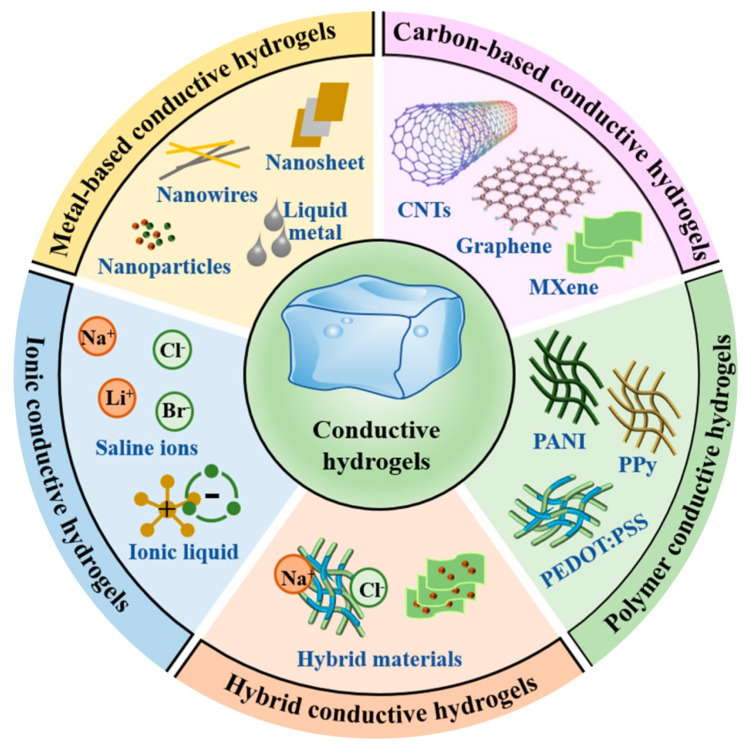
Classes of conductive materials used in electroconductive hydrogels. Adapted from ref. [100].

Electroconductive hydrogels have been a topic of research interest since their appearance in 1995 [12]. These biomaterials are created by introducing conducting elements into conventional hydrogel networks, some of which are highlighted in Figure 4. Among the most reported fillers, we have π-conjugated polymers, such as PPy and PANI, as well as carbon-based nanostructures, such as graphene and CNTs [12,13]. The delocalized electron clouds of these materials constitute pathways for charge transport within the 3D scaffold. Electroconductive hydrogels combine the high porosity and cytocompatibility of conventional hydrogels with the electrical responsiveness of their conductive components. Besides their responsiveness, the conductive fractions often improve the strength, resilience, and stability of the hydrogels, as well as induce cell attachment and spreading [13].

While numerous strategies exist to impart electroconductivity into hydrogels for a wide range of applications, ranging from platforms for biosensors and wearables [96], electrical-responsive drug release and wound healing stimulation, to tissue engineering [13], the present section specifically focuses on materials and methods employed in the context of wound healing. For broader overviews of conductive hydrogels in diverse biomedical fields, readers are referred to other comprehensive reviews [97,98,99].

### 4.1. Conducting Polymers

Conducting polymers (CPs) such as PPy, polythiophene, PEDOT, PANI, and polyacetylene are composed of conjugated chains that contain π-electrons that migrate along their single and double bonds [101]. These materials have a wide range of conductivities, from 10^−1^ to 10^7^ S·m^−1^, that can be harnessed to integrate them into various platforms with therapeutic potential [102,103], particularly in wound care applications [104]. When these polymers are placed in an aqueous environment, they provide hydrogels with much faster conductivity, due to electron transport, when compared to ion conduction [105]. This feature is crucial for their use in applications that require fast bio-electrical coupling. Nevertheless, these polymers carry some problems, such as the need for a high concentration of polymer to attain the percolation threshold—the concentration at which the material possesses a continuous and ubiquitous network of conductive pathways, in which electrons can travel unimpeded [106]. To face this issue, researchers often resort to agents that are simultaneously dopants and crosslinkers, which results in the lowering of the concentration required to achieve conduction [106]. Another problem that arises with these materials is their low degradability in vivo, since they remain in the tissue long after healing is complete. This is often addressed by grafting short aniline or pyrrole oligomers to biodegradable backbones, although this drastically decreases the conductivity of the final construct [104].

Lee et al. [107] developed a 3D-printable methacrylated gelatin (GelMA) hydrogel with microgrooved topology for wound healing. To endow their constructs with electrical conductivity, they blended a PEDOT:PSS solution with the GelMA hydrogel precursor. The digital light processing (DLP)-obtained constructs with a higher content of PEDOT:PSS (0.04% (*v*/*v*), termed P4, showed the best results: an impressive swelling capacity of 1692%, a tensile strength of 45 kPa, a conductivity 2.14 times higher than pristine GelMA, and a degradation profile that reached a 47.9% weight loss by day 28 of the experiment. Additionally, it was observed that an increase in PEDOT:PSS concentration caused a decrease in the hydrophilicity of the material’s surface. Human dermal fibroblasts (HDFs) proliferation and fibroblast growth factor 2 (FGF2), COL I, and matrix metalloproteinase 9 (MMP9) expression increased with PEDOT:PSS content and with external electrical stimulation (ES). FGF2 is related to the acceleration of wound healing, COL I regulates the secretion of collagen I, and MMP9 is involved in keratinocyte migration and, as such, are good metrics of wound healing. In vivo, a rabbit wound model exhibited a more regular reepithelialization, a higher density of fibroblasts, and enhanced collagen I deposition when treated with HDF-containing P4 hydrogel and ES. This study also serves as a pertinent example of how 3D printing can be applied to structure electroconductive hydrogels with precise topographical features that improve both electrical performance and biological activity. The DLP-based printing contributed to the creation of aligned grooves that mimic ECM cues and to improved wound closure outcomes in a rabbit model, as well. A recent review on 3D printing-based hydrogels for wound healing applications is available for a more detailed overview on this fabrication approach [108].

A similar approach, albeit with relevant differences, was attempted by Li’s team [109]. In their work, they devised a one-pot method in which they combined gelatin, dopamine, 3,4-ethylenedioxythiophene (EDOT), and PEG diglycidylether (PEGDE) to form a Gel-polydopamine (PDA)/EDOT emulsion that, afterwards, was polymerized, originating Gel-PDA/PEDOT hydrogels. According to the authors, this fabrication strategy allows for circumventing the issue of the poor dispersibility of these nanomaterials. The obtained hydrogel exhibited a conductivity of 68.7 S·m^−1^, a tensile strength of 132 kPa at 250% tensile strain, and a compressive strength of 3.8 MPa. Compared with the previous work [107], this hydrogel also showed a much lower swelling capacity (around 150%), which is attributed to the higher crosslinking density caused by the introduction of PDA into the system. The hydrogel also possessed shape memory and evidenced a complete enzymatic degradation after 15 days, which is much faster than the one developed by Lee et al. [107]. Gel-PDA/PEDOT scaffolds’ extract also promoted much higher L929 fibroblast migration in vitro and showed a capacity of scavenging ROS of over 65% in a DPPH assay. The antioxidant activity was attributed to the creation of a dynamic redox microenvironment within the construct, owing to the presence of PDA and PEDOT. Finally, an in vitro scratch assay evidenced superior migrational performance in the PEDOT-containing hydrogels.

PPy is one of the most studied CPs, since it possesses electrically conductive features, high stability, and absorbance properties around the near infra-red (NIR) region, which makes it predominantly useful in the synthesis of conductive hydrogels. In this context, Gan and colleagues [110] synthesized a hydrogel based on PPy nanorods (NRs) in situ formed within a PAM/CS interpenetrating polymer network. The hydrogel showed a high conductivity of 0.3 S·m^−1^ and outstanding mechanical properties (compressive strength of 6.5 MPa, compression modulus of 136.3 MPa, and stretchability > 250%). The hydrogel was prepared by UV photopolymerization, followed by absorption of pyrrole monomers and polymerization with FeCl_3_, in which Fe^3+^ ions chelate CS and consequently firmly fix the structure through CS-metal coordination crosslinking, which increases mechanical stability. In vitro tests showed the ability of the hydrogel to adhere, proliferate, and elongate C2C12 muscle myoblast cells, especially under 300 mV ES. Implanted in rat skin wounds, in vivo, the material induced faster closure of the wounds (healing ratio of 95.4% after 21 days compared to controls) and enhanced tissue regeneration with the generation of epithelial tissue, blood vessels, and hair follicles, especially when loaded with epidermal growth factor (EGF).

Aiming at diabetic wound repair in combination with muscle function assessment, Tang et al. [111] developed a hydrogel composed of *Astragalus membranaceus* polysaccharide (OA), CMCS, and sodium alginate (SA). In this blend, they introduced composite NPs made from PDA, PPy, and MnO_2_ loaded with resveratrol, a phenolic compound with strong antioxidant and anti-inflammatory properties. This hydrogel—termed COSGP@R—was obtained after testing several ratios of SA:CMCS:OA, with the 1:2:2 ratio possessing the best mechanical properties: compressive strength of 35 kPa, breaking strain of 67.5%, and maximum yield strain of 733.3%. Furthermore, qualitative data also evidenced the adhesive properties of COSGP@R to the fingers’ joints. The concentration of NPs of 2 mg/mL originated the hydrogels with the highest conductivity, of around 0.9 S·m^−1^. Additionally, the release of resveratrol was more prolonged and sustained at the acidic conditions of diabetic wounds, showing its potential therapeutic effect under these conditions. Hemolysis ratios were found to be within the international standard of under 5%, and cytocompatibility was proven through the use of L929 fibroblasts cultured in the hydrogels’ extract. Human umbilical vein endothelial cell (HUVEC) tube formation abilities were also shown to be improved upon culturing with the COSGP@R hydrogel. In a rat paw wound model, COSGP@R also decreased the wound area to just 1.1% at day 21, while decreasing expression of IL-6 and TNF-α, increasing that of VEGF and CD31, and increasing collagen deposition. Additionally, the hydrogel showed good ROS scavenging capabilities and could record electromyographic (EMG) signals—electrical activity of muscles—with a superior signal-to-noise ratio than commercially available electrodes.

In a study conducted by Lu’s team [112], a 3-sulfopropyl methacrylate (3SPMA) containing poly(2-hydroxyethyl methacrylate) (pHEMA), copolymerized with PPy, was developed to produce a conductive hydrogel with stable electrical conductivity in physiological environments. The hydrogel matrix is composed of methacrylate monomers and methyltrimethoxysilane (MTMS) as the crosslinker, and 3SPMA is used as an in situ dopant to keep the conducting performance. The as-prepared HM-PPy hydrogel possessed improved conductivity compared to the matrix hydrogel, which remained constant for seven days in Phosphate Buffered Saline (PBS), thanks to the covalent doping method. The hydrogel featured a swelling ratio 40% higher than the non-conductive matrix, mechanical flexibility with lower Young’s modulus, and higher tensile strain following the introduction of PPy. In vitro, the HM-PPy hydrogel showed a marked decrease in protein absorption (70% lower than commercial Hydrosorb^®^ - Paul Hartmann AG, Germany) and bacterial adhesion (93% lower for *S. aureus*) and high biocompatibility tested by cytotoxicity. HM-PPy conductive hydrogel-assisted ES (5 V, 40 Hz AC, 1 h) improved the migration of fibroblasts in the scratch assay more than the electrode-mediated ES, and a sustained enhancement of migration was observed. In vivo studies using diabetic rats with chronic wounds indicated that ES through the hydrogel promoted wound closure, a thicker epithelium, and more vascularized dermis, better than both electrode-based ES and hydrogel alone.

A multifunctional conductive hydrogel (PACPH) based on PDA-modified Ag NPs (AgNPs@PDA), cellulose nanocrystals/PPy (CNC/PPy) composites, and PVA was developed as a wound dressing to effectively treat diabetic wounds in the work of Guan et al. [113]. PACPH revealed high tensile stress up to 192 kPa, strain of 200%, and toughness of 14.5 kJ·m^−3^, strong tissue adhesion based on catechol groups in PDA, broad-spectrum antibacterial activity primarily by synergistic action of PDA and AgNPs, and improved conductivity through the CNC/PPy composite, enabling effective ES. In vitro, PACPH allowed the proliferation of NIH/3T3 fibroblasts and exhibited an almost 100% antibacterial ability against *S. aureus* and *E. coli*. In vivo results using diabetic rats full-thickness skin defect models showed that the integrated PACPH/ES treatment could effectively improve the wound healing rate, almost healed the wound on day 15, accompanied with a larger amount of collagen deposition, upregulated angiogenesis (CD31 expression), immunomodulation of macrophage polarization toward M2 phenotype, reduced inflammation (lower TNF-α expression), and increased neuroregeneration.

Multifunctional collagen-based hydrogels (CHLYs) were developed by Lin et al. [114], introducing cysteine-modified ε-poly(l-lysine) (ε-PL-SH), conferring inherent and broad-spectrum antibacterial activity and in situ-polymerized PPy NPs, responsible for CHLY free radical scavenging ability and good electroactivity. CHLY hydrogels showed a low swelling ratio, good compressive strength, and viscoelasticity due to chemical crosslinking, chelation, physical interaction, and nanoreinforcements in the matrix network. In addition, the material presented excellent tissue adhesion ability, low cytotoxicity, enhanced cell migration ability, and good blood coagulation performance, without causing hemolysis. CHLY hydrogels showed advantages in alleviating persistent inflammatory response, as well as promoting angiogenesis, epidermis regeneration, and collagen deposition at the wound sites, consequently accelerating full-thickness wound healing.

To investigate the ability of electroconductive hydrogels to treat severely infected wounds, Xiao et al. [115] fabricated a PVA/gelatin hydrogel crosslinked with phytic acid (PA)-functionalized PANI, and whose pores were loaded with Ag NPs for antibacterial purposes—calling it Ag NPs/CPH. The optimized formulation had a conductivity within the dermis range—0.54 S·m^−1^—which was high enough to light a small lightbulb. Swelling capacity was measured, and it was observed that, after 72 h, it stabilized at around 160% for the Ag NPs containing hydrogel and around 180% for its counterpart without NPs, which indicates the ability to manage wound exudate. The microporous structure also promoted sustained Ag^+^ release that caused dose-dependent inhibition zones against *S. aureus* and *E. coli*. In liquid cultures, bacteria’s OD fell markedly as the dose of Ag NPs increased. Cytotoxicity assays with the hydrogel’s extract showed less than 20% of HaCat, LO2, and 293T cell death when the concentration did not exceed 150 μg/mL. In vivo, Ag NPs/CPH hydrogel led to a decrease in mice infected wound area by day 14 to 10%, when compared to the 40% in the gauze-treated group. Finally, histological results showed a decrease by half of inflammatory cells by day 7, double the number of blood vessels of the controls, and improved collagen deposition. All these observations demonstrated the potential of this material to decrease infection through Ag^+^-mediated sterilization and accelerate healing. Wu and colleagues [116] also explored the use of PANI as an antibacterial and conductive agent. PANI and sulfonated hyaluronic acid as a dopant were used to construct a flexible hydrogel with skin-mimicking electrical conductivity. In vivo results demonstrated that ES (DC, 5 V, 75 Hz, 30 min for 3 days) through this hydrogel was superior to ES via separated electrodes for promoting infected chronic wound healing.

### 4.2. Carbon Nanomaterials

The electrical conductivity of carbon-based nanomaterials is derived from a continuous and delocalized network of π-electrons that allows them to move freely through overlapping orbitals, originating constructs with enhanced conductivity [117]. These materials include CNTs, graphene and its derivatives, and fullerenes. Despite their superior conductive performance, they lack proper solubility in water, which limits their applications within hydrogels [118]. To address these issues, one of the strategies employed is through chemical functionalization. This approach improves colloidal stability but comes at the expense of a decrease in conductivity due to perturbations in the conjugated structure [118]. As is the case for conductive polymers mentioned earlier, carbon-based nanomaterials linger in the body after healing due to their poor degradability [117]. As such, new 2D biodegradable materials are being explored to counteract this effect, such as black phosphorus or MXene (Ti_3_C_2_T*x*) nanosheets—a family of 2D materials composed of transition metal carbides, nitrides, or carbonitrides [117].

Fan et al. [119] showed how graphene can enhance the bioactivity of hydrogels. Hydrogels were synthesized by crosslinking silver (Ag)/graphene (G) with acrylic acid and N,N′-methylene bisacrylamide, using glucose as a reducing agent. This material, Ag5G1, with a 5:1 Ag-to-graphene ratio, combined enhanced mechanical strength, porous structure, and high swelling capacity (~1456%) due to the presence of graphene, with the antibacterial activity of Ag, while maintaining low cytotoxicity in fibroblasts. In vivo studies carried out on rats using full-thickness skin wounds indicated that the Ag5G1 hydrogel accelerated wound closure, achieving up to 98% wound closure in 15 days, accompanied by histological evidence of minimized inflammation, promoted fibroblast migration, collagen deposition, and full and thickened epidermis formation. According to the study of Zmejkoski et al. [120], graphene can improve the antibacterial activity of hydrogels. The hydrogel composite that was produced by loading bacterial cellulose (BC) with ~11.7 wt% graphene quantum dots (GQDs) was found to be biocompatible, as well as to have antibacterial properties against a large range of bacteria. It has also been demonstrated that this graphene-based hydrogel facilitates wound healing by improving water retention and fluid absorption, as well as by encouraging human fibroblast migration and angiogenesis.

CNTs incorporation in hydrogels is twofold beneficial to wound healing applications, since they enhance the mechanical properties of the hydrogel matrix and allow for the transmission of electrical signals around the wound area, which enhances the healing process of the wound and tissue regeneration through improved cell migration and blood flow [121]. Biologically, CNTs enhance the therapeutic performance of the hydrogels by creating favorable interactions with cells, biomolecules, and native tissue that enhance cellular migration within the hydrogel [122]. Liang et al. [121] demonstrated the benefits of the incorporation of CNTs in the wound healing process. In their study, PDA was used to coat the surface of the CNTs (CNT-PDA), to reduce the hydrophobic interactions of CNTs. CNT-PDA and gelatin-grafted-dopamine (GT-DA) were used to synthesize GT-DA/CS/CNT composite hydrogels via the oxidative coupling of catechol groups using a H_2_O_2_/horseradish peroxidase (HRP) catalytic system. The study showed that the conductivity of the hydrogels rose from 2.5 × 10^−2^ S·m^−1^ to 7.2 × 10^−2^ S·m^−1^ as the CNTs concentration in the hydrogel increased from 0 wt% to 4 wt%. These results are within the reported range for human skin conductivity, which lies between 1 × 10^−5^ and 0.26 S·m^−1^ [123]. Additionally, when the CNT-laden hydrogel was applied to an infected full-thickness mouse skin defect wound, antibacterial, antioxidant, adhesive, wound closure, and collagen deposition properties were observed.

Liu et al. [124] used MWCNTs to provide a CS and gelatin blend with electroconductive properties, coupling it with skin repair. The mixture was crosslinked and freeze-dried to render it highly porous, a feature that was shown not to be destroyed by the presence of the MWCNTs. The conductive fillers increased wet conductivity from 2.42 × 10^−2^ S·m^−1^ to the around 2 × 10^−1^ S·m^−1^, with the formulation with the highest concentration of MWCNTs (0.5 wt%) showing improved mechanical performance—13.7 kPa and 54 kPa of tensile and compressive stresses, respectively, and a compressive modulus of 15 kPa—while rendering the hydrogel’s surface more hydrophobic and lowering the degradation rate. However, the 0.3 wt% MWCNTs formulation was selected as the optimal concentration via cellular assays, since human skin fibroblasts viability was the highest and cell spreading was observed. Additionally, moderate ES (1 V, 30 min) further improved proliferation, directed migration as a response to the electrical field, and higher expression of COL1A1, COL3A1, and fibronectin 1 (FN1)—factors associated with skin regeneration—was achieved when compared to the controls that were not stimulated. Nevertheless, this assay did not show results in vivo and, as such, further validation is still required.

MXenes have received significant attention in biomedical applications due to their hydrophilicity, excellent electrical conductivity, mechanical flexibility, high biocompatibility, and high antibacterial activity. These features render MXene an outstanding nanofiller for the synthesis of conductive hydrogels [117].

Aiming at simultaneous diabetic wound healing and monitoring, Liu et al. [125] developed a zwitterionic poly(acrylamide-co-sulfobetaine methacrylate) (P(AM-co-SBMA)) matrix with embedded PDA-functionalized MXene sheets that were combined with Ag NPs (PMAg), creating hydrogels termed PPMAg. PDA is introduced into the system as a tool to avoid aggregation of the MXene nanosheets. PMAg improved the stretchability of the hydrogels and, from all the tested formulations, PPMAg-0.75 delivered the best solution, stretching to 2020% at a tensile strength of 117 kPa, toughness of 993 kJ m^−3,^ and a Young’s modulus in the range of muscle’s at 10 kPa. Furthermore, it showed tight adhesiveness to pig skin (8.5 kPa), without leaving residues on it upon peeling. This material also evidenced a conductivity of 3.7 × 10^−2^ S·m^−1^, owing to the MXene/Ag structure that created electronic pathways. In vitro, PPMAg-0.75 maintained L929 fibroblasts viability over 90% and elicited approximately 99% of *S. aureus* and *E. coli* death within 4 h. After being placed in a diabetic rat wound and acting in conjunction with an external electrical stimulus, the hydrogel diminished the wound area by 77% by day 7, as opposed to only a 28% decrease in controls. This material also proved effective in the detection of rat pulse, as well as bending signals from the knee, finger, and elbow, with significant sensitivity in distinguishing types of bending from the former joint. These results showcase its potential as a platform for these two purposes in combination with wound healing.

Diabetic ulcers were also the focus of the work by Wang et al. [123]. The researchers developed a hydrogel by polymerizing GelMA with MXene nanosheets, which they then strengthened through the introduction of tannic acid (TA), that formed H-bonds with GelMA chains. The optimal formulation, termed GMT, had a concentration of MXene of 400 μg·mL^−1^, and evidenced skin-like conductivity (1.9 × 10^−1^ S·m^−1^); a Young’s modulus of around 156 kPa, which falls in the range of the dermis’s (88–300 kPa); and high compressive strength of 818 kPa. Additionally, the catechol chemical features of the TA led to good adhesiveness of the produced hydrogel to several surfaces—plastic, metal, glass, paper, rubber, and skin tissue—with testing showing an adhesive strength of 17 kPa to porcine skin, which was 2.8 times higher than the pristine GelMA hydrogel. The hydrogel also possessed a strong antioxidant response, scavenging over 90% of DPPH and three different biologically relevant ROS (H_2_O_2_, ∙OH, and ∙O_2_^−^), through the action of the TA polyphenols that preferably react with infiltrating water and oxygen, consuming oxidative agents. This antioxidant role was also confirmed with an L929 fibroblast in vitro assay. Migration also improved with this hydrogel, with an in vitro assay showing 60% closure within 12 h vs. 45% in the controls. In a full-thickness diabetic wound model, GMT promoted 92% of wound closure by day 14, while simultaneously decreasing by half the expression of IL-1β, an inflammatory agent, and tripling and doubling CD31 and VEGF expression, respectively. On top of that, a thick and tight collagen matrix was also observed in the GMT-treated group—77% vs. 65% in the non-MXene treated group—showing the potential of the synergy between MXene electroactivity and TA-mediated redox action to accelerate diabetic wound regeneration.

### 4.3. Noble Metal Nanomaterials

Noble-metal NPs—gold (Au) or Ag, for example—are also used to endow hydrogels with superior electrical properties due to thermodynamic diffusion and random collision between NPs [126]. These metallic particles can also be engineered in 1D shapes, such as NRs or nanowires, which are able to decrease the percolation threshold. The good conductivity and antibacterial properties of these materials render them promising for wound healing applications [127].

Ag^+^ ions, in particular, are quite attractive for research in electroconductive hydrogels. In addition to the previous studies already described that combine CPs or carbon nanomaterials with Ag NPs in the synthesis of conductive hydrogels for wound healing [113,115,125], Zheng et al. [128] devised a β-CD-modified PVA hydrogel, combined with β-CD-encapsulated Ag NPs (^CD^AgNPs) and β-glucan functionalized with hyaluronic acid (HAG). This material presented a porous structure that was deemed to facilitate exudate absorption and cell adhesion at wound sites. In terms of swelling ratio, it was shown that ^CD^AgNPs promoted an increase in this metric, possibly due to osmotic pressure. PVA_15_/CD/HAG/^CD^AgNP hydrogel evidenced excellent adhesive properties to pig skin (11.6–13 kPa vs. 5 kPa for a commercial fibrin glue used as control), ideal for wound dressings. The measured conductivity was 8.6 × 10^−2^ S·m^−1^, 5.5 times higher than its counterpart without ^CD^AgNP. PVA_15_/CD/HAG/^CD^AgNP ([^CD^AgNP] = 1.2 μg·mL^−1^) also promoted the death of 99.99% of *E. coli* and *S. aureus* within 1 h and 4 h, respectively. After its extract showed cytocompatibility in NIH 3T3 fibroblasts in vitro, with proliferation increasing further upon ES, wounds were inflicted in a rat model. It was observed that they were closed within 6 days when exposed to ES, while also showing decreased inflammation, a higher number of hair follicles, higher collagen density, and minimal scarring. Furthermore, it also showed good hemostatic activity. Comparing this work with a previously cited one [115], it is possible to observe the value of combining different strategies to obtain the desired outcomes, as well as the importance of fine-tuning the design of the hydrogels: for instance, this work reports a higher wound closure rate—6 days to full closure vs. 10% of wound area by day 14. However, it reports a lower conductivity—8.6 × 10^−2^ S·m^−1^ vs. 0.54 S·m^−1^.

In other work [129], Ag NPs were combined with lignin (a nanomaterial with nontoxic, biodegradable, and antimicrobial properties) and introduced in combination with quercetin-modified melanin NPs within a CS and SA dual-network hydrogel—termed CALQ—to promote diabetic ulcer healing and motion monitoring. Firstly, the authors optimized the CS:SA ratio through rheological measurements, reaching the conclusion that the one suitable for following experiments would be 1:1, since it yielded more viscous materials and higher yield strain (1199%). Subsequently, the optimal concentration of the lignin-Ag NPs was found to be 8% due to improved mechanical performance, for example, improved elongation at break when compared to human skin. Finally, rheological assessments of the CALQ hydrogel exhibited shear-thinning behavior and better maximum strain when compared to its counterpart without quercetin-melanin NPs. The conductivity of the CALQ hydrogel was the same as the one reported in the previous study (8.6 × 10^−2^ S·m^−1^), albeit possessing lower swelling potential (30% when compared to the 396% of the PVA hydrogel). A promising feature of this hydrogel consists of its pH-responsive release of quercetin, which responded to an acidic environment such as the one present in diabetic ulcers. At a 4 h timepoint, the hydrogel caused 81% and 94% of *E. coli* and *S. aureus* death, respectively, and it also did not show hemolytic activity. In vivo, the wound area was drastically reduced to around 20% by day 14, with decreased expression of IL-6 and TNF-α, increased expression of VEGF and CD31, and a higher granulation tissue density. Finally, the hydrogel was able to detect several body movements, such as finger bending, smiling, swallowing, pressing, wrist bending, nodding, and frowning, showing its potential to be used in the biosensor field, as well.

Although explored to a much lower extent, gold has also been utilized in the preparation of conductive hydrogels with wound healing potential. For instance, Au NPs were prepared and used in the synthesis of conductive hydrogels of PEG–AuNR and cationic poly(allylamine) hydrochloride (PAH)-AuNRs [130]. These hydrogels were observed to show enhanced wound healing properties (enhanced skin re-epithelialization, collagen deposition after 14 days, and anti-inflammatory response) using an animal model, and in vitro antibacterial properties against *S. aureus* and *P. aeruginosa*. These gold-based conductive hydrogels, therefore, presented a promising platform for future skin wound healing applications.

Of note, although several conductive fillers have been researched, their effects on wound healing are usually assessed in vastly different models, hindering cross-comparison. There is also a lack of systematic studies looking at electrical conductivity level as a variable and its impact on biological outcomes (e.g., inflammation or angiogenesis). The optimal conductivity range for therapeutic purposes is still unclear.

## 5. Self-Healing, Electroconductive Hydrogels: Wound Healing Evidence

Having explored the mechanisms behind self-healing and conductivity separately, this section now examines how both features can be integrated into a single platform for wound healing.

Self-healing electroconductive hydrogels have grown from laboratory research interests to multifunctional wound dressings able to handle the daily movements of patients while furnishing wounds with the electrical, biochemical, and antimicrobial cues that accelerate tissue repair. Recent literature has shown how the combination of polymeric networks with conductive elements can, indeed, mimic skin tissue and actively promote healing at cell and tissue levels. These materials have shown better performance than current gold-standard wound dressings in treating wounds, such as Tegaderm™, since they have been shown to promote improved wound closure with blood vessels and have epidermal thickness and skin appendages closer to human skin, with decreased inflammation, as well [38,65,95].

Table 1 summarizes the existent studies applying hydrogels integrating both conductivity and self-healing properties for wound treatment.

Zhao et al. [38] started by grafting 4-formylbenzoic acid-rich PEG-co-poly(glycerol sebacate) (PEGS)—PEGS-FA—to QCS and combining these with PANI as the conductive agent. The hydrogel showed rapid self-healing properties owing to the imine bond formation, and PANI provided the hydrogel with increased antioxidant properties and skin-like conductivities. Furthermore, it was shown that an increase in PEGS-FA concentration led to a decrease in conductivity, gelation time, and pore size while increasing the swelling ratio. This material also showed a critical strain of 250% and recovery properties. This platform showed no hemolytic activity against erythrocytes, no decrease in cell viability of cells cultured with media conditioned with the hydrogel, and hydrogels with a PEGS-FA concentration over 1% showed almost total resistance against E. coli and S. aureus. In vivo, QCSP3/PEGS-FA1.5 hydrogel showed hemostatic activity, prevented blood loss, and promoted a higher wound contraction rate while also improving granulation tissue thickness and increasing expression of VEGF, EGF, and TGF-β [38].

Building on the self-healing and antimicrobial foundation established by the previous study, Zhang and colleagues [90] also used QCS as a structural base, developing a host–guest-based self-healing hydrogel by functionalizing it with β-CD and adamantane (AD), exploiting the matching degree of β-CD and AD to reach a strong host–guest interaction. Additionally, the authors used reduced graphene oxide (rGO) grafted with β-CD to also interact with the AD-functionalized QCS chains. In terms of the properties of the hydrogel, it was noted that an increase in rGO concentration caused a decrease in the storage modulus and increases in the pore size and photothermal response. Additionally, the material showed good adhesiveness properties to the skin, increasing with rGO content from 110 Pa (0 wt% rGO) to 173 Pa in the formulation with the highest concentration (0.6 wt% rGO). Finally, the hydrogels also showed shear-thinning behavior and complete recovery of properties after being subjected to high strains. In accordance with what was observed in the previously mentioned study, this biomaterial also showed antimicrobial activity against *E. coli* and *S. aureus*, and also against Methicillin-resistant *S. aureus* (*MRSA*), which increased with an increase in rGO concentration and irradiation time. Hemolytic activity was verified for rGO concentrations above 0.4 wt%, and therefore, higher concentrations were not used in further cell studies. It was shown that rGO promoted the proliferation of L929 fibroblasts, and in vivo studies showed accelerated wound closure and increased collagen content and fibroblast number, with lower levels of inflammatory cells. Finally, rGO-treated groups also showed lower levels of IL-6 and higher levels of VEGF, indicating lower inflammation and improved potential for angiogenesis.

**Figure 5:**
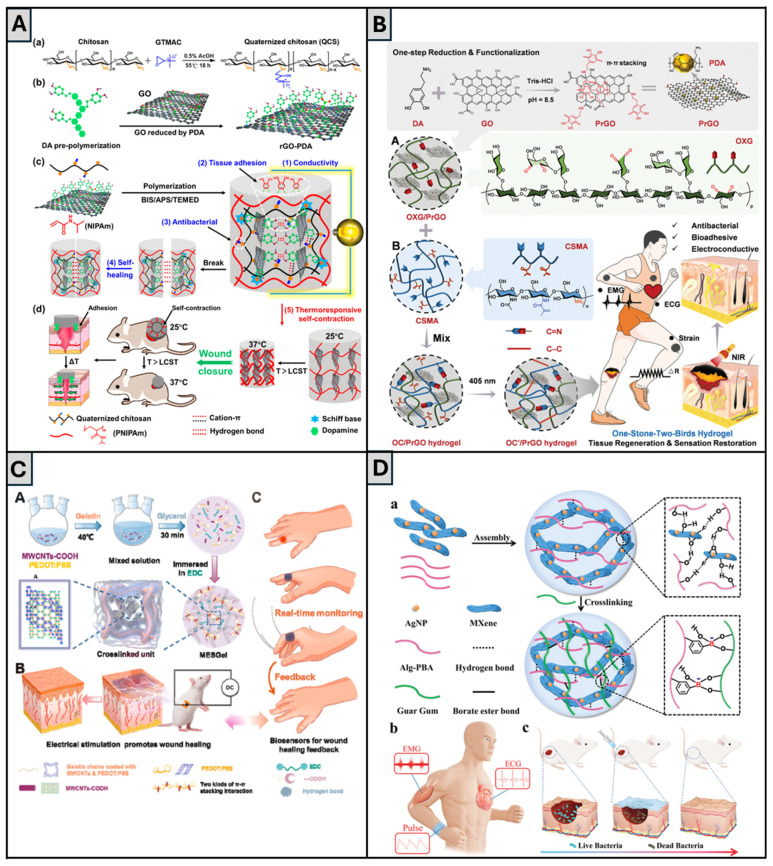
Schematic representation of (**A**) (a) Synthetic route of QCS, (b) Preparation and properties of rGO-PDA, (c) QCS/rGO-PDA/PNIPAm hydrogel, (d) wound closure by thermoresponsive self-contraction of the hydrogel. Reprinted with permission from ref. [66]. Copyright 2020 American Chemical Society. (**B**) Design of OC′/PrGO hydrogels for skin regeneration and mechanosensation restoration: (A) OXG/PrGO (PDA-modified rGO nanosheets/oxidized xyloglucan); (B) CSMA (methacrylate chitosan) Reproduced with permission from ref. [64] Copyright 2025 Wiley. (**C**) (A) Synthetic process of MESGel. (B) ES promoting wound healing. (C) Real-time monitoring and feedback of MESGel biosensors for wound healing. Reproduced with permission from ref. [131] Copyright 2021 Elsevier. (**D**) (a) fabrication of the healable, injectable, and antibacterial MXene hydrogel and further applications in (b) human healthcare monitoring and (c) wound therapy. Reproduced with permission from ref. [95] Copyright 2022 Wiley.

Li et al. [66] also relied on the use of QCS and rGO to create biomechanically active, injectable, self-healing, conductive, and adhesive hydrogels, with wound healing applications, composed of QCS, PDA-coated rGO (rGO-PDA), and poly(N-isopropylacrylamide) (PNIPAm) (Figure 5A). PNIPAm confers to the hydrogel its heat-sensitive shrinkage, contracting above the lower critical solution temperature (~33 °C), helping wound closure by exerting biomechanical forces. QCS adds strong tissue adhesion, along with antibacterial and hemostatic features. rGO-PDA boosts electrical conductivity, antioxidant activity, and photothermal antibacterial capability, and its catechol groups further strengthen tissue adhesion. Results from in vitro studies showed that the hydrogel had excellent self-healing ability, swelling behavior with temperature-dependent pore size reduction, as well as temperature-triggered release of the drug doxycycline. The hydrogels displayed conductivities of 0.42–0.56 S·m^−1^ (similar to human dermis—0.22 S·m^−1^ [66]) and possessed intrinsic antibacterial activity against *E. coli* and *S. aureus*, in particular, under NIR photothermal treatment. Hemocompatibility and cytocompatibility studies corroborated that the hydrogels were non-toxic. In vivo, a full-thickness skin wound model in mice demonstrated that QCS/rGO3-PDA/PNIPAm hydrogel stimulated wound contraction, with a wound area decrease of 83.6% vs. 74.6% in the control without rGO, and wound healing with increased granulation tissue thickness, collagen deposition, promoted vascularization (shown by CD31 expression), and reduced inflammation (lower IL-6 levels). The addition of doxycycline enhanced antibacterial activity and the progression of wound healing. It is worth pointing out that these researchers achieved encouraging results in terms of wound contraction in vivo while using a smaller concentration of rGO than the previously discussed work [90], which is positive, since rGO has a low degradation rate and can linger in the body after healing, potentially causing issues [133].

In a similar direction, Wei et al. [64] also employed PDA-modified rGO that was integrated within a photo-crosslinked hydrogel optimized for dynamic joints and motion detection (Figure 5B). The authors blended oxidized xyloglucan and methacrylate CS, with the incorporation of PDA-modified rGO nanosheets (PrGOs), to prepare a dynamic hydrogel termed OC/PrGO. When compared to the control without PrGO, this hydrogel showed a slightly improved shear-thinning behavior and, unlike Zhang et al.’s report [94], increased storage and loss moduli. Its photo-crosslinked version, OC’/PrGO, was able to adhere to human and porcine skin without detachment due to the presence of PDA with its catechol groups. Nevertheless, its adhesive strength was lower than the one reported by Li et al. [66], who used a lower concentration of rGO than the one reported in this work, meaning that there may exist a detrimental effect of rGO content in the adhesive properties of hydrogels. This hydrogel could also detect motion in joints such as the knee, elbow, and finger. OC’/PrGO also promoted HUVEC migration at levels similar to the control group. Hemolysis was also tested and shown to be within the acceptable standards of <5%. Increases in PrGO concentration also caused an improvement in antibacterial behavior and increased photothermal response, as well. Finally, OC’/PrGO-treated mice models also showed a faster healing rate (84% vs. 63%) and a more native-like epidermal layer. These features underscore its applicability as a dressing for highly mobile anatomical regions.

Moving toward printable and highly modular formulations, Ali et al. [67] engineered a PEG diacrylate (PEGDA)-based hydrogel crosslinked with dithiothreitol (DTT), using borax as a catalyst. As conductive fillers, the authors explored the use of PEDOT:PSS, AuNPs, or MXene sheets that provided the material with self-healing properties via interaction with -SH groups, in the case of the AuNPs, or H-bonding through the -SO_3_H groups of PEDOT:PSS and -OH, -O, and -F groups in MXene sheets. The hydrogel showed good extrudability and the ability to recover its structural integrity and elasticity within 30 min at 37 °C after breaking under shear. The designed dressings also showed proper adhesiveness to skin and showed conductivities similar to native skin that increased with the concentration of filler. The material was responsive to glucose and pH, which could be used for controlled drug release, an important feature for the treatment of infected wounds or diabetic ulcers. In vitro tests demonstrated the biocompatibility and wound healing efficacy of the hydrogel, as it stimulated cell proliferation, closure of wounds, and had an antibacterial effect on common pathogens, possibly by the controlled, stimuli-triggered release of the antibiotic tetracycline hydrochloride (TCH). The use of different conductive fillers within the same hydrogel allows for drawing conclusions on the strengths of each one. The rheological properties of all were very similar, despite the PEDOT-containing hydrogel showing a slightly higher storage modulus and viscosity, with the opposite occurring in the AuNPs-containing one. In terms of conductivity, the AuNP-laden hydrogel showed lower values than its PEDOT:PSS and MXene counterparts, with the combination of these last two leading to the formation of a more conductive material than the remaining three. Finally, it was also observed that the hydrogel that was made with MXene had a higher bactericidal potential than its counterparts. These results highlight the need to choose the proper materials for the desired applications.

While the previous study emphasized the versatility of fabrication of hydrogels, Zheng et al. [131] turned to the material’s conductivity as an active stimulus. The authors developed MESGel, a gelatin network with self-healing properties owed to H-bonding, electrostatic interactions, and π–π stacking, in which they introduced PEDOT:PSS and carboxyl-functionalized MWCNTs (MWCNTs-COOH) to confer electroconductivity (Figure 5C). MESGel was able to withstand a tensile strain up to 425% and a compressive strain up to 71%, values that remained almost unaltered after self-healing (375% and 65%, respectively), and also showed a higher denaturation temperature than its non-conductive and PEDOT:PSS- or MWCNTs-only counterparts. It was also observed that a detection device based on MESGel allowed the detection of bending motion in joints such as the wrist, elbow, and finger, showing its potential to be used in monitoring movement in wounds in these regions. Finally, ES of the MESGel (DC, 100 mV/mm, 25 Hz, for 1 h, with 1 h of interval, followed by 1 h of stimulation, with intervals of 2 h afterwards) was proven to increase cell proliferation in vitro. In vivo experiments employing a full-thickness skin defect model in rats demonstrated that MESGel combined with ES accelerates wound closure (over 90% within 10 days) and promotes granulation tissue formation, collagen deposition, re-epithelialization, and angiogenesis, as shown by elevated expression of PDGF and VEGF. These results highlight the potential use of MESGel as both a sensor device and a therapy to improve wound healing.

Expanding on the concept of self-healing sensors, the research by Zhang et al. [91] also showed potential for use in wound healing and sensory devices. The authors used CMCS and PBA-grafted SA (Alg-PBA), which they combined with MXene nanosheets and tea polyphenols (TP). This material had self-healing properties due to the dynamic covalent borate ester bonds between Alg-PBA and the -OH groups of CMCS and TP, as well as the supramolecular interaction between all these components and MXene. The mechanical performance of these hydrogels improved with increases in the concentration of several components: PBA, TP, and (up to 3.0 mg/mL) MXene nanosheets. Furthermore, the hydrogel showed shear-thinning properties. Increase in the MXene concentration also increased the conductivity of the scaffolds; but when it exceeded the 3.0 mg/mL threshold, it reduced the sensitivity to bending of the hydrogel. This sensitivity to elbow bending was also found not to be attenuated after the hydrogel was cut and healed. The hydrogel showed good adhesiveness to porcine skin, withstanding up to three cycles of adhesion/peeling, which highlights its potential as a wound dressing, and that was attributed to the presence of TP within the structure. Interestingly, a hydrogel-integrated sensor also detected various body signals, such as swallowing, finger and elbow bending, wrist pulse, and ECG and EMG signals, showing its versatility in signal detection. In terms of cytocompatibility, conditioned media did not decrease cell viability, and the hydrogel showed hemocompatibility and bactericidal activity against *S. aureus* and *E. coli*. Additionally, in rat liver and tail wound models, the hydrogel drastically decreased blood loss.

Complementing the MXene/TP approach of the previous study, Ren et al. [132] created a GelMA-based hydrogel loaded with Ti_3_C_2_ MXene nanosheets and with a collagen-functionalized antimicrobial agent (V-Os), which originated an in situ photocrosslinkable matrix that the authors denominated GelMA@Ti_3_C_2_. The nanosheets originated a conductive hydrogel, with a conductivity that remained even after the hydrogel was cut and healed, while also improving the matrix’s stiffness to a tensile strength of approximately 190 kPa. V-Os and Ti_3_C_2_ conferred very effective bactericidal activity, as shown by the death rate of over 90% of *E. coli* and *S. aureus*, as well as by the almost sterile wounds verified in vivo. It is also worth noting that the functionalization with collagen was essential to improve binding to the GelMA network. Cytocompatibility was also observed, with the proliferation and adhesion of NIH-3T3 improving, as well as the expression of VEGF and COL I, when the scaffold was exposed to an external ES. In a rat model, 94% wound closure was observed by day 11 in the hydrogel and ES-treated group (square wave, 100 Hz, 200 mV), results that were better than those counterparts that did not contain MXene. Additionally, histology assays confirmed a denser deposition of collagen, improved angiogenesis—shown by CD31+ staining—and decreased TNF-α expression. Hence, this platform shows the potential to accelerate wound healing while killing any microorganisms that may affect recovery.

Introducing a different conductive backbone, Khan et al. [92] developed a PVA-based hydrogel, with Ag NPs-functionalized GO in the presence of thioglycolic acid (TGA)—GAT-PVA. In a different formulation, a PANI shell was formed around the GO compound, originating GATP-PVA hydrogels. These hydrogels had a self-healing ability by H-bonding and disulfide bonds between PVA chains, which allowed their storage modulus to remain nearly constant through three break cycles. The thixotropic and shear-thinning behavior of these materials was also rheologically verified. TGA was proven to improve hydrogel’s adhesion, a feature that is essential for wound healing. The GATP-PVA hydrogel also showed enhanced conductivity and antibacterial activity against *S. aureus*, *P. aeruginosa*, and *K. pneumoniae*. GAT and GATP nanocomposites improved L929 viability in vitro when compared to pristine PVA hydrogels, which shows they enhanced cytocompatibility. Finally, an in vivo wound model was treated with hydrogels for the assessment of hemostatic and wound healing properties. GATP-PVA hydrogel led to the lowest blood loss when the mouse was injured in its liver, as well as improved wound contraction (up to 90% on day 15). Additionally, the GATP-PVA-treated group exhibited denser collagen layers, more new blood vessels, and improved reepithelialization, showing its potential as a wound dressing.

In a related antibacterial framework, Qiao et al. [65] reported the development of a multifunctional antibacterial conductive self-healing hydrogel wound dressing. This material was composed of oxidized SA grafted with dopamine (OSD), CMCS, Fe^3+^ ions, and PDA-coated poly(thiophene-3-acetic acid) (PA), which established dual dynamic bonds via Schiff base and Fe^3+^ coordination, allowing excellent self-healing activity by automatic repair of the disrupted hydrogel network. The hydrogel showed excellent mechanical strength, antioxidant ability, strong tissue adhesion, hemostasis, and tunable rheological properties, with an increase in conductivity from 3.0 × 10^−4^ S·m^−1^ for OSD/CMCS hydrogel to 7.2 × 10^−4^ S·m^−1^ by adding PA and Fe^3+^, which promoted wound healing through enhanced electrical signaling. Photothermal antibacterial function upon NIR irradiation led to more than 99% killing of *MRSA* in 5 min and 100% eradication of *E. coli* within 10 min. In vivo studies in a full-thickness mouse skin wound with *MRSA* infection showed that the group of mice treated with OSD/CMCS/Fe/PA3 hydrogel that received NIR treatment displayed significantly accelerated wound healing, with its ratio reaching 97.02% after 14 days, compared with commercial Tegaderm™ film, as well as hydrogels without PA or NIR. The histological analysis showed a decrease in inflammation and an increase in the thickness of granulation tissue, in addition to more angiogenic activity (higher level of VEGF) and skin appendages as a result of hydrogel decreasing pro-inflammatory TNF-α and promoting tissue regeneration.

In another study, Zhao et al. [93] prepared an injectable double-network hydrogel composed of xanthan gum (XG), dopamine-modified oxidized SA (OSA-DA), fucoidan, and bioglass (45S5) for effective full-thickness wound healing and health monitoring. The first network of the hydrogel is constructed by hydrogen bonding between XG chains. The second network results from chelation between Ca^2+^ ions leaching from the bioglass and the carboxyl groups of OSA, reinforced by Schiff base linkages with dopamine that enhance tissue adhesion. The hydrogel system showed remarkable biomechanical features: it adhered strongly to a range of surfaces (highest adhesion to porcine skin stems from catechol groups and Ca^2+^ ion chelation), self-healed within 30 min, and showed shear-thinning injectability suitable for filling irregular wounds. It released bioactive ions and fucoidan, encouraging angiogenesis, collagen deposition, and epithelial regeneration. In vitro tests on NIH 3T3 fibroblasts confirmed good biocompatibility, with proliferation exceeding 300% and migration rates rising sharply. The gel also presented antibacterial effects against *E. coli* and *S. aureus*, with inhibition rates of 40% and 32%, respectively. Hemolysis assays revealed blood compatibility. In vivo, when placed on full-thickness wounds in Sprague Dawley rats, the hydrogel closed wounds almost completely by day 9, outperforming commercial 3M dressings and controls. Tissue sections revealed denser collagen fibers, more blood vessels (translated by higher VEGF and CD31 signals), and milder inflammation (shown by lower TNF-α levels). Additionally, the ions present imparted electrical conductivity (0.65 S·m^−1^), allowing the hydrogel to function as a flexible biosensor that tracks in real time large joint movements, fine facial gestures, voice emissions, and pulse signals.

Building on the idea of integrating natural polysaccharides with electroconductive agents, Li et al. [14] created a GG-based hydrogel, termed guar slime (GS), which they combined with PEDOT:PSS (PPGS). It was observed that PPGS showed double the conductivity of the pristine GS hydrogel and an increased strain critical point (500% vs. 150% for GS), albeit with a lower storage modulus (150 Pa vs. 400 Pa). Finally, the hydrogel showed thixotropic behavior, highlighting its self-healing properties that were attributed to the H-bond between hydroxyl groups in GS. GS- and PPGS-extracts did not show cytotoxicity against three different cell types—Madin–Darby canine kidney (MDCK), human aortic fibroblasts (HF), and 3T3 cells. The hydrogels also did not show relevant hemolytic activity and led to an enhanced healing rate in vitro, especially PPGS, with 71% vs. 57% for GS at day 7. In vivo, the hydrogels were applied in high-motion areas of rats (back of the head and dorsum), with the PPGS-treated group showing higher granulation tissue thickness, collagen deposition, and hair follicle regeneration. Hence, this material has great promise for use in wound healing applications.

A highly stretchable, conductive, and self-healing hydrogel based on PVA crosslinked with PPy-functionalized CS (CS-PPy) and zinc-chelated CS (ZnCS) was developed by Zhang et al. [94]. The network was formed through dynamic di-diol complexations, hydrogen bonding and zinc-based coordination bonds, which conferred the hydrogel, named PCPZ, high mechanical robustness (high stretchability ~3500% without fracture) and rapid autonomous self-healing (70 min, without applying external stimuli). With an electrical conductivity of 116 S·m^−1^ due to the presence of the PPy moieties, it functions as a temperature and strain sensor for finger movements, with reliable signal reproducibility, even after the cutting and healing process. The biocompatibility was demonstrated through cytotoxicity and hemolysis tests, results of which yielded no harm to fibroblasts and erythrocytes. Combined with ZnCS, the resultant hydrogel possessed excellent antibacterial activity. In vivo evaluation in diabetic rats with infected chronic wounds revealed that the ES (DC, 3 V for 1 h per day, for 3 days) of the PCPZ hydrogel, compared to controls or hydrogel treatment alone, led to a much faster wound healing rate, promoting tissue regeneration and angiogenesis.

Li and colleagues [95] developed a MXene-based hydrogel composed of antimicrobial Ag NPs-coated MXene (AgNPs/MXene) nanosheets incorporated into a polymeric network through GG and Alg-PBA (Figure 5D). It was found that the hydrogel possessed high mechanical strength, superior electrical conductivity associated with the conductive AgNPs/MXene nanosheets network, and excellent antibacterial capability resulting from the effect that the Ag NPs can disrupt the bacterial membrane and interfere with the bacterial metabolism. The self-healing performance is attributed to dynamic crosslinking among -OH groups in GG and PBA groups in Alg-PBA, as well as supramolecular interactions of AgNPs/MXene, GG, and Alg-PBA, resulting in the fast restoration of mechanical and electrical properties after damage. The injectable hydrogel also exhibited excellent shear-thinning properties and degraded up to 45 days in PBS. It was demonstrated that the hydrogel had no cytotoxicity to L929 cells, according to the in vitro cytocompatibility tests, and the bactericidal ratios were 77.78% and 85.82% against *S. aureus* and *E. coli*, respectively. In a murine full-thickness wound model infected with *S. aureus*, the hydrogel significantly promoted wound closure to 98.16% in 12 days, as compared to the control. Furthermore, the MXene hydrogel was successfully constructed as a multifunctional skin sensor, which can sensitively record full-scale human motions and tiny electrophysiological signals, including ECG and EMG, with higher SNR compared to commercial electrodes.

The evidence laid out in this section shows the multitude of materials that can be used for wound healing purposes, highlighting the importance of electroconductivity to improve wound healing properties, and also showing the need and efficacy of self-healing materials in high-motional areas of the body while keeping their multifunctionality. Many published in vivo studies concentrate only on short-term endpoints (e.g., 7 to 14 days), which creates gaps in understanding the long-term tissue integration, immune responses, or the chronic impacts of conductive degradation byproducts. A more detailed analysis of these aspects is provided in Section 6. In addition, most models are rodent-based, and more extensive studies involving larger animals or human skin equivalents are extremely limited.

## 6. Translational Challenges and Future Steps

Electroconductive self-healing hydrogels have emerged as one of the most promising materials to be used in advanced wound dressings, since they match the current trend in the biomedical field of not only covering the wounds, but also modulating and accelerating wound healing by interacting with their microenvironment [134]. As such, and as was shown in this review, extensive work has been put into the development of these biomaterials, with quite promising results.

These materials have intrinsically high water content and porosity, which allows them to manage the exudate of the wounds [16] while at the same time maintaining a gas-permeable interface between the wound and the external environment [135]. Coupling these materials with electroconductive fillers allows the recreation of endogenous electrical fields that originate upon injury, which guide fibroblasts and keratinocytes migration to promote healing in a way that conventional dressings cannot recreate [136]. This is particularly relevant for wounds that cannot heal on their own, since the exogenous electric fields generated by applying ES in the presence of these conductive materials can assist the treatment.

Finally, developing materials with specific crosslinking features can endow them with self-healing features, providing them with the ability to heal cracks that can form due to natural body movements, making them ideal for use in high-motion areas such as the joints [137] while reducing patient discomfort and dressing change frequency [138]. Often, these features are also combined with antibacterial agents that can suppress bacterial proliferation—and, consequently, infections—and/or scavenge ROS, further improving wound closure, since this also reduces inflammation [7]. In contrast, current clinical gold-standard dressings (e.g., gauze, films, foams, and hydrocolloids) primarily provide passive wound coverage and suffer from several limitations, including frequent replacement, poor conformability to irregular wound geometries, and lack of active biological functionality [139]. Therefore, the studies presented in this review highlight the transformative potential of such materials to outperform traditional wound dressings both mechanically and biologically; nevertheless, no direct clinical comparisons have yet been made.

Nevertheless, the transition from academically interesting materials to practically useful wound dressings remains complex. Achieving an adequate trade-off between mechanical and conductive properties is still challenging, and it may be difficult to conjugate the material’s conductive components with the hydrophilic polymer network of the hydrogel when one exceeds a certain concentration [140], which may be necessary for monitoring purposes. Long-term stability of the hydrogels can also be a concern that can be partially circumvented by providing them with self-healing properties; but there are still problems concerning the degradability issues of conductive fillers in biological environments [141]. It has been reported that carbon and metal nanomaterials [136], as well as conductive polymers [142], have poor degradability in vivo, which can cause potential long-term toxicity. One example is the potential accumulation of MXene in the lungs, increasing the risk of pulmonary dysfunction [143], while there have also been reports on the ability of GO to penetrate into the dermis, which can be indicative of accumulation on the skin [144]. Similarly, CPs such as PEDOT:PSS are not biodegradable in physiological environments and may prompt inflammatory responses upon prolonged implantation [145]. Recent efforts have been directed toward addressing these challenges by formulating biodegradable conductive materials, namely, CPs (e.g., ester-linked hybrids of PANI or PPy with polylactide or CS) that offer a combination of electroactivity with controlled enzymatic degradation and enhanced biocompatibility [146], which may offer safer profiles for long-term applications. Despite this, these strategies remain at the early stages of preclinical evaluation.

In in vivo studies, ES is typically applied using wearable patches or electrode interfaces in contact with the hydrogel dressing [147]. However, the ES parameters that have been reported are highly heterogeneous: current densities, stimulation durations ranging from 30 min to several hours daily, and both DC and pulsed waveforms being employed. The only common factor found was the stimulation intensity, which was found within or very close to the values concerning endogenous electrical fields (40–200 mV). This makes sense, since one of the objectives is the replication of these fields to accelerate wound healing; but the applications of ES may also be related to the triggered release of healing- accelerating substances, among others. To translate these materials into clinical use, there is a clear need for the standardization of protocols and deeper understanding of the optimal ES regimens for therapeutic efficacy [148].

Given the diverse nature of wounds, which can range from diabetic ulcers to easier-to-treat surgical incisions, it is challenging to establish standardized protocols for the use of bioelectronic biomaterials. There is a need to guarantee consistent results across patient populations and decrease the risks that patients may be exposed to. Furthermore, there is a lack of uniformity across the regulatory bodies that handle this type of device, which can hinder their global availability, ultimately preventing certain geographic regions from accessing this type of treatment. Finally, integrating these materials into the existing wound care pipeline also remains difficult [149].

One of the most investigated uses for electroconductive hydrogels is their application in wearables and soft electronics due to their excellent conductivities, mechanical properties, adhesion, and biocompatibility [150]. These hydrogels allow real-time monitoring of wound status, resorting to several different biomarkers, such as pH and temperature, while also providing drug delivery on demand [151]. However, some constraints for their widespread use remain: the collection of high-quality data is still difficult, as well as the establishment of medical data platforms [136] that can use the data to drive decision-making. Additionally, the integration between conductive hydrogels and microelectronic devices is still difficult, since the inherent properties of the hydrogels, such as high water content and flexibility, render their connection with the rigid and dry electronic components very difficult, and the long-term stability of the hydrogels is also a concern [152]. As such, the development of these materials is a multidisciplinary job, requiring collaboration among clinicians, material designers, electronics specialists, and IT experts.

The rise of artificial intelligence (AI) can also have a profound impact on the design of these hydrogels. Diagnostic platforms integrating deep learning algorithms enable clinicians to monitor the progress of wounds in real time and adapt the therapeutic approach to enhance healing [153]. AI-driven simulations can also contribute to material design by allowing researchers to test new materials in silico. By predicting the properties of a hydrogel, such as swelling behavior, mechanical strength, and biocompatibility, it is possible to greatly reduce resources and time spent on its development, accelerating the entire formulation process [154].

The irregular and dynamic nature of wounds demand adaptable solutions, and 4D-printed hydrogels could serve as an attractive strategy to this challenge [155]. Constructed using stimulus-responsive materials, these systems are able to change shape and stiffness or function in response to particular stimuli such as pH, temperature, or light [155,156]. A multifunctional 4D hydrogel dressing was recently prepared employing thermo-responsive PNIPAm and curcumin-loaded Pluronic micelles, with PEGDA-dopamine serving as a degradable adhesive crosslinker. This structure exhibited excellent hemostasis, tissue adhesion, anti-*MRSA* capabilities, and inflammation modulation to promote diabetic rat wound closure [157]. Beyond temperature-sensitive performance, 4D hydrogels also enable on-demand antimicrobial release through embedded triggers, which can further improve the responsiveness for smart wound dressings [155]. Additionally, natural polymers, including chitosan, have been introduced into 4D-printed structures due to their biocompatibility, intrinsic antimicrobial activity, and enhanced ability to mediate cell adhesion [156]. Leveraging 4D methodologies, these fabricated chitosan composites can be designed based on patient-specific wound topographies, as well as wound healing dynamics, thus contributing to targeted precision skin regeneration. Integration with AI algorithms can then enhance this process, allowing the scaffold architecture and the therapeutic payload to be tailored for optimal tissue regeneration [155]. However, there are various challenges that should be resolved before 4D-printed constructs are used in the clinical wound care setting, including regulatory validation, long-term safety, and large-scale manufacturing.

Finally, there is also the need to optimize the animal models used to test these dressings. Each animal species has its own set of wound healing characteristics that may not be directly translatable to humans: pigs have a similar skin architecture to humans, making them suitable to study reepithelialization processes, whereas rabbits are a more suitable model to investigate ischemia, for instance [158]. Therefore, it is crucial to choose the right animal model to represent the features that the test is trying to replicate, to avoid downstream issues that can have an impact on the waste of resources or, ultimately, on human health.

Overall, electroconductive self-healing hydrogels combine electroconductivity, mechanical resilience, adhesiveness, and antibacterial properties, offering a strong alternative to traditional wound dressings. However, they encounter several key translational challenges. Conductive nanomaterials’ long-term safety analysis in wound healing environments requires scalable, Good Manufacturing Practices (GMP)-compliant synthesis methods. Standardized preclinical systems with comprehensive metric frameworks would enable efficient cross-study comparisons. Furthermore, there is a striking lack of clinical trials. Nevertheless, there is a prospective opportunity for their translation into clinical applications, which will depend on their scalability and integration with biosensors and AI-assisted design, bringing additional challenges, including the ergonomic power source and overall patient usability. Lastly, cost-effectiveness must be carefully evaluated to justify their adoption over conventional wound dressings. Once these challenges are met, which requires interdisciplinary collaboration, these hydrogels can change the wound dressing paradigm.

## 7. Conclusions

Self-healing electroconductive hydrogels represent a blend of polymer chemistry, bioelectronics, and regenerative medicine that is revolutionizing the wound care world. Through the integration of self-healing properties with electronic pathways provided by CPs, carbon nanomaterials, MXenes, or metallic nanocrystals, researchers have shown that the creation of constructs that are simultaneously moist, mechanically resilient, self-healing, and conformable to irregular wound shapes is possible. These materials can actively interfere with the microenvironment of wounds, accelerating reepithelialization, improving the migration of cells such as fibroblasts, stimulating angiogenesis, and decreasing the risk of bacterial infections. The electroconductivity of the hydrogels tested in these works also showed, in most cases, to be in the range of the native dermis, which restores the endogenous currents that are created upon injury, incentivizing healing. Furthermore, we also reported on the fabrication of materials able to control inflammation and infection on demand, through the remotely controllable release of antibiotics and/or antioxidants. The current challenges and future perspectives on this growing field were also laid out.

At the end of the day, the success of this technology is heavily dependent on multidisciplinary collaboration. Materials engineers must achieve a fine balance among mechanical performance, conductivity, and cytocompatibility; biomedical engineers must participate as experts in integrating these materials with flexible circuitry and wireless technology to allow monitoring of wounds; clinicians must ensure that the developed materials serve their desired purpose—faster wound closure, reduced scar tissue, restoration of appendages, all while keeping patient discomfort at a minimum; and regulatory specialists must guide the manufacturing process to comply with the ever-evolving standards. After all these specifications are assured, the next generation of wound dressings will not only protect damaged skin tissue, but also actively participate in its regeneration.

## Figures and Tables

**Table 1:** State-of-the-art on electroconductive, self-healing hydrogels for wound healing applications.

Hydrogel Composition	Conductive Components	Self-Healing Mechanism	Physico-Chemical Performance	Biological Outcomes	Ref.
QCS-PANI (QCSP) and PEGS-FA	PANI	Dynamic Schiff base bonding between QCSP amines and PEGS-FA aldehydes; network reinforced by π–π stacking and electrostatic interactions	Conductivity: optimal 0.237 S·m^−1^ (QCSP3/PEGS-FA1.5) Gelation: 86–374 s; G’: 58–368 Pa; Swelling: 170–200%; Pore size: 107–204 µm; Critical strain: 250%; Thixotropic; Excellent antioxidant activity (>84% DPPH scavenging) improved with PANI; Adhesiveness: up to 4.9 kPa	In vitro: >95% viability (L929), non-hemolytic, >99% kill (*E. coli*, *S. aureus*); In vivo: mouse full-thickness wound model; 10% better contraction at day 10 vs. Tegaderm™; ↑ collagen, granulation, EGF, TGF-β, VEGF; ↓ inflammation	[38]
QCS-β-CD, QCS-AD, GO-β-CD	GO-CD	Dynamic host–guest interactions (β-CD and AD) + hydrogen bonding between QCS-β-CD, QCS-AD, GO-β-CD	Conductivity: 0.07–0.11 S·m^−1^ (GO4); Swelling ratio ~113%; Adhesive strength: 130 Pa; Shear-thinning and injectable; Photothermal ΔT: 20.1°C (10 min, 808 nm, 1.4 W/cm^2^); Network recovered within 14 s after strain-induced collapse	In vitro: L929 fibroblast proliferation ↑ with rGO; Hemolysis < 5%; Antibacterial: 95% *E. coli* death (2 h), 100% *S. aureus* and *MRSA*; NIR photothermal effect: complete kill in 10 min. In vivo (mice full-thickness wounds): 98.3% wound closure (Day 14); ↑ collagen, VEGF, epidermal/granulation tissue thickness; ↓ IL-6	[90]
QCS, rGO-PDA, PNIPAm, BIS, APS/TEMED	rGO-PDA	Dynamic Schiff base bonds between PDA and QCS; cation–π interactions; non-covalent interactions between catechol (PDA) groups; hydrogel reforms after strain cycles and incubation at 25 °C for 2 h	Self-healing strain threshold: 611%; Recovery of G′ and G″ after 1000% strain; Conductivity: up to 0.56 S·m^−1^ with rGO-PDA 4 mg/mL; Swelling ratio (25 °C): 436–588%, decreases to 205–302% at 37 °C; Tensile stress: up to 148.9 kPa after 60 min self-contraction at 37 °C; Adhesion to porcine skin: 9.68 kPa	In vitro L929 fibroblasts. Antibacterial: >85% killing (*E. coli, S. aureus*); 100% kill under NIR (10 min); DPPH scavenging ~97.8%; L929 viability: 82–93%; hemolysis < 2%; Doxy release: 93% over 10 days at 37 °C; In vivo: full-thickness skin defect in mice; comparison with commercial Tegaderm. Contraction-assisted closure: wound size reduced to 49 ± 6% on day 7; Histology: ↑ COL (76%), ↑ granulation, ↑ CD31, ↓ IL-6, ↑ hair follicles (865%)	[66]
OXG (oxidized xyloglucan), CSMA, PrGO	PDA-modified rGO nanosheets (PrGO)	Dynamic Schiff base bonds between aldehyde groups (OXG) and amine groups (CSMA)	Conductivity: 0.17 S·m^−1^; Modulus: from ~100 Pa (dynamic) to 20 kPa (after UV-triggered crosslinking); Adhesive strength to porcine skin: 4.6 kPa; NIR-induced photothermal conversion: ΔT ≈ 20.2 °C; Max surface temp: ~47 °C; DPPH scavenging: 77.8%; Potential to detect motion in different joints (knee, elbow, finger)	In vitro: HUVECs—↑ viability (106% at 24 h), ↑ migration (scratch assay), Hemolysis ratio < 5%; In vivo: full-thickness wound in ICR mice—Day 5: 83.9% closure (vs. 63.0% control), Day 21: full re-epithelialization, ↑ granulation (199 μm), ↑ COL (81%), ↑ immune regulation (↑ Th17/Th1/Th2 pathways) Hydrogel-treated group showed faster healing rate (84% vs. 63%) and a more native-like epidermal layer	[64]
PEGDA (matrix), DTT (crosslinker), Borax (catalyst)	AuNPs, PEDOT:PSS and/or MXene	Thiol-acrylate Michael addition forms covalent PEGDA–DTT network; DTT provides –SH groups; Borax facilitates reversible boronate ester bonds with DTT diols	3D-printable, injectable; Stretchability ~210%; G′ up to 417.5 Pa (P:P/MX-HG); Conductivity (S·m^−1^): 2.36 (pHG), 159.3 (AuNP-HG), 229.0 (P:P-HG), 229.5 (MX-HG), 283.6 (P:P/MX-HG); Adhesiveness to skin; Self-healed hydrogels (within 30 min at 37°C) retain G′ and conductivity; pH responsive, adequate for drug release in infected wound conditions.	In Vitro, HDF-Ad cells: ↑ viability over 7 days; Scratch wound assay: full closure at 24 h with pHG, TCH-HG; slower initial closure for P:P/MX-HG; Antibacterial effect (inhibition zones): 22.3 mm (*E. coli*), 20.8 mm (*P. aeruginosa*);	[67]
Gelatin with EDC + PEDOT:PSS + MWCNTs-COOH (MESGel)	PEDOT:PSS and MWCNTs-COOH	Hydrogen bonding, electrostatic interactions, and π–π stacking among gelatin, PEDOT:PSS, and MWCNTs-COOH	Conductivity: 0.71 S·m^−1^ (2.0 mL PEDOT:PSS); Rapid self-repair (60% in 2 min; ~100% in 10 min); Stability retained post self-healing; Tensile strain: 425% (pre)/375% (post-healing); Compressive strain: 71%/65%; Swelling ratio > 4.5; Thermal denaturation temp: 65.1°C; Shear-thinning; Broad viscoelastic range; Degradation: 92.3% in 3 wks; Potential to detect motion in different joints (wrist, elbow, finger)	In vitro: CHL cells—viability > 111.3%, CPI: 63.7 (vs. 59.1 MESGel), enhanced by ES; In vivo: rat full-thickness skin model—>90% wound closure (day 10), ↑ collagen, granulation, re-epithelialization, PDGF, VEGF; high sensor performance (ΔR/R_0_ > 40%) and 100 ms response time in joint motion monitoring	[131]
CMCS, Alg-PBA, TP, MXene nanosheets	MXene nanosheets	Dynamic borate ester bonds between -OH groups (CMCS, TP) and PBA (Alg-PBA); supramolecular interactions (hydrogen bonding, π–π stacking, cation–π) between CMCS, TP, MXene, and Alg-PBA	Conductivity: improved with MXene, but excessive MXene content led to decreased sensitivity to bending; Stretchability: up to 300%; Adhesion strength: 32.76 kPa (plastic), 18.74 kPa (porcine skin); Self-healing: seconds (ambient conditions); Injectable; Degradation: ~21 days in PBS; Sensing: GF = 0.79 (vs. 0.26 without MXene), unchanged in the cut-and-healed hydrogel; Detected swallowing movements, finger and elbow bending and wrist pulse, as well as ECG and EMG signals	In vitro: L929 fibroblasts—no cytotoxicity (72 h); Hemolysis: minimal (comparable to PBS); Antibacterial: 93.06% (*S. aureus*), 96.30% (*E. coli*). In vivo: Mouse liver hemorrhage and tail amputation models—↓ blood loss (30.66 mg (liver) vs. 446.64 mg (control), 18.76 mg (tail) vs. 265.54 mg (control)); EMG and ECG signals monitored effectively during motion; SNR (EMG): 29.7 dB vs. 16.7 dB (commercial electrode)	[91]
GelMA, Ti_3_C_2_ MXene, V-Os (collagen-binding antimicrobial peptide)	Ti_3_C_2_ MXene	Physical self-healing through reversible supramolecular interactions between GelMA chains, Ti_3_C_2_ MXene nanosheets, and the collagen-binding peptide V-Os	Conductivity: 0.7 mS m^−1^; Tensile 0.193 MPa; Porosity 89%; Swelling 351%; Injectable	In vitro: NIH3T3—↑proliferation and adhesion under ES (100 Hz, 200 mV), ↑COL-I and VEGF expression; Bacterial survival ≈ 0%; In vivo: full-thickness rat wound model—wound closure 94% (day 11, ES); ↓ TNF-α, ↑ CD31 angiogenesis, ↑ COL-I; ↓ scar tissue	[132]
PVA (matrix), GO/Ag/TGA nanocomposites, PANI (optional shell)	GO/Ag/TGA; with or without PANI coating (GATP-PVA)	Hydrogen bonding between PVA chains; TGA introduces thiol and carboxyl groups that enhance hydrogen bonding and interfacial adhesion; In GATP-PVA, disulfide bonding and π–π interactions with PANI shell further stabilize network	Shear-thinning and thixotropic behaviors; Tensile strength ~1.1 MPa; Conductivity (ionic): 0.138 S·m^−1^ (GATP-PVA); Self-healing demonstrated via oscillatory rheology over 3 break–heal cycles with minimal G′ loss; Strong tissue adhesion attributed to TGA-functionalized GO;	In vitro L929 fibroblasts: ↑ viability vs. pristine PVA; ROS scavenging properties In vivo mouse wound model: ~90% wound contraction at day 15 with GATP-PVA; Histology: denser collagen, ↑ vascularization, ↑ reepithelialization; Hemostatic: ↓ liver bleeding volume in GATP group; Antibacterial: strong activity vs. *S. aureus*, *P. aeruginosa*, *K. pneumoniae*	[92]
OSD, CMC, Fe^3+^, and PDA coated poly(thiophene-3-acetic acid))	PDA-coated poly(thiophene-3-acetic acid) (PA)	Dual dynamic bonding: (1) Schiff base between aldehyde (OSD) and amino groups (CMC); (2) Metal coordination between catechol (OSD/PA) and Fe^3+^	Swelling ratio: 240% (OSD/CMC/Fe/PA5); Degradation: ~63 h; Conductivity: 7.2 × 10^−2^ S·m^−1^ (PA5); ΔT (photothermal): 25°C @10 min NIR; G′: 120.9 Pa (PA5); Adhesive strength > 5 kPa (pigskin test)	In vitro: L929 cells (viability ≥ 80%); Antibacterial: *E. coli* and *MRSA* (99% in 5–10 min NIR); In vivo: mouse full-thickness *MRSA*-infected wound model; wound closure 97.02% (Day 14, PA3 + NIR); ↓ TNF-α, ↑ VEGF and angiogenesis	[65]
XG, OSA-DA, fucoidan, and 45S5 bioglass microspheres	Ca^2+^ and Si^4+^ ions released from 45S5 bioglass	Dynamic Schiff base bonds between dopamine amines and OSA aldehydes; enhanced by hydrogen bonding (XG), Ca^2+^ chelation (OSA), and π–π stacking from catechol groups	Conductivity: 0.65 S·m^−1^ (vs. 0.4 S·m^−1^ in groups without bioglass); Adhesion: >12 kPa on porcine skin; Swelling ~350% at 48 h; Shear-thinning; G’ recovery after 200% strain; Porous 3D structure; Fucoidan release sustained > 120 h	In vitro: NIH 3T3 proliferation (>300% RGR), migration (scratch and Transwell), hemolysis < 2%, antibacterial (*E. coli*: 40%, *S. aureus*: 32% inhibition); In vivo: SD rat full-thickness wound model, 98% wound closure by day 9, ↑ VEGF/CD31/collagen, ↓ TNF-α	[93]
GG-based slime (GS) (matrix)	PEDOT:PSS	Dynamic intermolecular hydrogen bonding between –OH groups in CG chains; PEDOT:PSS electrostatically interacts with CG quaternary ammonium groups; gel reforms within 30 min at RT without external stimulus.	Injectable; Stretchability up to 500% (vs. 150% for GG alone); Conductivity: 0.22 S·m^−1^ (vs. 0.104 S·m^−1^ in CG alone); Both hydrogels showed thixotropic behavior	No cytotoxicity in MDCK, HF, and 3T3 cells; hemolysis < 5%; In vitro wound healing: closure rate 71% (PPGS) vs. 57% (GG) on day 7; In vivo (rat, dorsum, and occiput wounds): Histology: ↑ granulation tissue, 76% COL deposition (vs. 56% CG, 20% control); ↓ inflammation, ↑ hair follicle regeneration.	[14]
PVA, Zn^2+^-functionalized CS (ZnCS), CS-PPy, borax (crosslinker)	CS-PPy	Combination of reversible di-diol complexation (PVA-borax), Zn^2+^-CS coordination bonds, and hydrogen bonding	Conductivity: 116 S·m^−1^; Tensile stretchability: >3500%; Self-healing time: 10 s; Stable after autoclaving and freezing; LED circuit reconnection validated	In vitro: fibroblasts—no cytotoxicity; Hemolysis < 2%; Antimicrobial against *S. aureus* and *P. aeruginosa* In vivo: diabetic infected rat model—↑ wound closure in PCPZ + ES (3 V, 1 h/day); ↑ COL deposition, re-epithelialization, and mature blood vessels on day 21	[94]
GG, Alg-PBA, AgNPs-coated MXene nanosheets, NaOH	AgNPs/MXene nanosheets	Dynamic crosslinking between –OH in GG and PBA groups in Alg-PBA; supramolecular interactions among AgNPs/MXene, GG, and Alg-PBA	Tensile strain: 166.67% (original) vs. 165.28% (healed); Break strength recovery: ~95%; Shear-thinning; Injectable; Degrades in 45 days in PBS (pH 7.2); 3D porous structure with high hydrophilicity	In vitro: L929 fibroblasts—no cytotoxicity; Antibacterial efficacy: 77.78% *(S. aureus*), 85.82% (*E. coli*); Epidermic sensor detects wrist/finger bending, swallowing, ECG, EMG (SNR: 17.8 dB); In vivo: murine full-thickness infected wound (8 mm)—wound closure 98.16% (day 12) vs. 80.5% (control); ↓ inflammation, ↑ collagen, ↑ vascularization	[95]

↑ symbolizes increase/improvement; ↓ symbolizes decrease/reduction

## Data Availability

No new data were created or analyzed in this study. Data sharing is not applicable to this article.

## Abbreviations

The following abbreviations are used in this manuscript:

3SPMA	3-sulfopropyl methacrylate
AC	Alternating Current
AD	Adamantane
AI	Artificial Intelligence
Ag	Silver
Alg-PBA	Phenylboronic acid-grafted Sodium Alginate
Au	Gold
BC	Bacterial Cellulose
bFGF	Basic Fibroblast Growth Factor
CD	Cyclodextrin
CMCS	Carboxymethyl Chitosan
CNTs	Carbon Nanotubes
CPs	Conducting Polymers
CS	Chitosan
DLP	Digital Light Processing
DPPH	2,2-Diphenyl-1-picrylhydrazyl
DTT	Dithiothreitol
ECG	Electrocardiogram
EDOT	3,4-Ethylenedioxythiophene
EGF	Epidermal Growth Factor
EMG	Electromyogram
EMT	Epithelial-to-Mesenchymal Transition
ES	Electrical Stimulation
FGF	Fibroblast Growth Factor
FN1	Fibronectin 1
GG	Guar Gum
GMP	Good Manufacturing Practice
GQDs	Graphene Quantum Dots
GelMA	Methacrylated Gelatin
HDF	Human Dermal Fibroblast
HRP	Horseradish Peroxidase
HUVEC	Human Umbilical Vein Endothelial Cell
IL	Interleukin
MDCK	Madin–Darby Canine Kidney
MMP9	Matrix Metalloproteinase 9
*MRSA*	Methicillin-resistant *Staphylococcus aureus*
MTMS	Methyltrimethoxysilane
MWCNTs	Multi-Walled Carbon Nanotubes
NIR	Near Infrared
NPs	Nanoparticles
NRs	Nanorods
NT3	Neurotrophin 3
OA	Oxidized Alginate
PA	Poly(thiophene-3-acetic acid)
PAA	Poly(acrylic acid)
PACPH	Polydopamine/AgNPs/Cellulose Nanocrystals/Polypyrrole Hydrogel
PAM	Polyacrylamide
PANI	Polyaniline
PBA	Phenylboronic Acid
PBS	Phosphate-Buffered Saline
PDA	Polydopamine
PEDOT	Poly(3,4-ethylenedioxythiophene)
PSS	Polystyrene Sulfonate
PEG	Poly(ethylene glycol)
PEGDA	Poly(ethylene glycol) Diacrylate
PEGDE	Poly(ethylene glycol) Diglycidyl Ether
PEGS	Polyethylene Glycol-co-poly(glycerol sebacate)
PNIPAm	Poly(N-isopropylacrylamide)
PPy	Polypyrrole
PrGO	Polydopamine-modified Reduced Graphene Oxide
rGO	Reduced Graphene Oxide
ROS	Reactive Oxygen Species
RT	Room Temperature
SA	Sodium Alginate
SBMA	Sulfobetaine Methacrylate
SNR	Signal-to-Noise Ratio
TA	Tannic Acid
TCH	Tetracycline Hydrochloride
TGA	Thioglycolic Acid
TNF-α	Tumor Necrosis Factor Alpha
TP	Tea Polyphenol
UV	Ultraviolet
VEGF	Vascular Endothelial Growth Factor
XG	Xanthan Gum
ZnCS	Zinc-chelated Chitosan

## References

[1] Yazdi S.J.M., Baqersad J. (2022). Mechanical Modeling and Characterization of Human Skin: A Review. J. Biomech..

[2] Peña O.A., Martin P. (2024). Cellular and Molecular Mechanisms of Skin Wound Healing. Nat. Rev. Mol. Cell Biol..

[3] Wilkinson H.N., Hardman M.J. (2020). Wound Healing: Cellular Mechanisms and Pathological Outcomes. Open Biol..

[4] Li Y.-Y., Ji S.-F., Fu X.-B., Jiang Y.-F., Sun X.-Y. (2024). Biomaterial-Based Mechanical Regulation Facilitates Scarless Wound Healing with Functional Skin Appendage Regeneration. Mil. Med. Res..

[5] Hassanshahi A., Moradzad M., Ghalamkari S., Fadaei M., Cowin A.J., Hassanshahi M. (2022). Macrophage-Mediated Inflammation in Skin Wound Healing. Cells.

[6] Falanga V., Isseroff R.R., Soulika A.M., Romanelli M., Margolis D., Kapp S., Granick M., Harding K. (2022). Chronic Wounds. Nat. Rev. Dis. Primer.

[7] Liang Y., He J., Guo B. (2021). Functional Hydrogels as Wound Dressing to Enhance Wound Healing. ACS Nano.

[8] Ghomi E.R., Niazi M., Ramakrishna S. (2023). The Evolution of Wound Dressings: From Traditional to Smart Dressings. Polym. Adv. Technol..

[9] Koehler J., Brandl F.P., Goepferich A.M. (2018). Hydrogel Wound Dressings for Bioactive Treatment of Acute and Chronic Wounds. Eur. Polym. J..

[10] Almeida D., Sanjuan-Alberte P., Silva J.C., Ferreira F.C. (2023). 3D (Bio)Printing of Magnetic Hydrogels: Formulation and Applications in Tissue Engineering. Int. J. Bioprinting.

[11] Gounden V., Singh M. (2024). Hydrogels and Wound Healing: Current and Future Prospects. Gels.

[12] Lu H., Zhang N., Ma M. (2019). Electroconductive Hydrogels for Biomedical Applications. WIREs Nanomed. Nanobiotechnol..

[13] Rogers Z.J., Zeevi M.P., Koppes R., Bencherif S.A. (2020). Electroconductive Hydrogels for Tissue Engineering: Current Status and Future Perspectives. Bioelectricity.

[14] Li S., Wang L., Zheng W., Yang G., Jiang X. (2020). Rapid Fabrication of Self-Healing, Conductive, and Injectable Gel as Dressings for Healing Wounds in Stretchable Parts of the Body. Adv. Funct. Mater..

[15] Dhivya S., Padma V.V., Santhini E. (2015). Wound Dressings—A Review. BioMedicine.

[16] Abd-ElSalam H.-A.H., Refaeey O.A., Waked K.G., Elsherbiny K.A., Aleam A.M., Ibrahim M.Q., Farag M.H., Nasef A.M., ElMeshad A.N. (2024). A Review Exploring the Wound-Healing Activity of Self-Healing Hydrogels: Fabrication, Characterization, Mechanism, and Biomedical Applications. J. Clust. Sci..

[17] Talebian S., Mehrali M., Taebnia N., Pennisi C.P., Kadumudi F.B., Foroughi J., Hasany M., Nikkhah M., Akbari M., Orive G. (2019). Self-Healing Hydrogels: The Next Paradigm Shift in Tissue Engineering?. Adv. Sci..

[18] Jia N., Yang J., Liu J., Zhang J. (2021). Electric Field: A Key Signal in Wound Healing. Chin. J. Plast. Reconstr. Surg..

[19] Mamun A.A., Shao C., Geng P., Wang S., Xiao J. (2024). Recent Advances in Molecular Mechanisms of Skin Wound Healing and Its Treatments. Front. Immunol..

[20] Lu C., Kolbenschlag J., Nüssler A.K., Ehnert S., McCaig C.D., Čebron U., Daigeler A., Prahm C. (2021). Direct Current Electrical Fields Improve Experimental Wound Healing by Activation of Cytokine Secretion and Erk1/2 Pathway Stimulation. Life.

[21] Raja R. (2007). Wound Re-Epithelialization: Modulating Kerationcyte Migration in Wound Healing. Front. Biosci..

[22] Yan T., Jiang X., Guo X., Chen W., Tang D., Zhang J., Zhang X., Zhang D., Zhang Q., Jia J. (2017). Electric Field-Induced Suppression of PTEN Drives Epithelial-to-Mesenchymal Transition via mTORC1 Activation. J. Dermatol. Sci..

[23] Xue L., An R., Zhao J., Qiu M., Wang Z., Ren H., Yu D., Zhu X. (2025). Self-Healing Hydrogels: Mechanisms and Biomedical Applications. MedComm.

[24] Li Z., Lu J., Ji T., Xue Y., Zhao L., Zhao K., Jia B., Wang B., Wang J., Zhang S. (2024). Self-Healing Hydrogel Bioelectronics. Adv. Mater..

[25] Xu L., Chen Y., Yu M., Hou M., Gong G., Tan H., Li N., Xu J. (2023). NIR Light-Induced Rapid Self-Healing Hydrogel toward Multifunctional Applications in Sensing. Nano Energy.

[26] de Luna M.S., Marturano V., Manganelli M., Santillo C., Ambrogi V., Filippone G., Cerruti P. (2020). Light-Responsive and Self-Healing Behavior of Azobenzene-Based Supramolecular Hydrogels. J. Colloid Interface Sci..

[27] Cao J., Zhang D., Zhou Y., Zhang Q., Wu S. (2022). Controlling Properties and Functions of Polymer Gels Using Photochemical Reactions. Macromol. Rapid Commun..

[28] Habault D., Zhang H., Zhao Y. (2013). Light-Triggered Self-Healing and Shape-Memory Polymers. Chem. Soc. Rev..

[29] Huang W.-C., Ali F., Zhao J., Rhee K., Mou C., Bettinger C.J. (2017). Ultrasound-Mediated Self-Healing Hydrogels Based on Tunable Metal–Organic Bonding. Biomacromolecules.

[30] Amaral A.J.R., Pasparakis G. (2017). Stimuli Responsive Self-Healing Polymers: Gels, Elastomers and Membranes. Polym. Chem..

[31] Zhang Y., Fu C., Li Y., Wang K., Wang X., Wei Y., Tao L. (2017). Synthesis of an Injectable, Self-Healable and Dual Responsive Hydrogel for Drug Delivery and 3D Cell Cultivation. Polym. Chem..

[32] Deng C.C., Brooks W.L.A., Abboud K.A., Sumerlin B.S. (2015). Boronic Acid-Based Hydrogels Undergo Self-Healing at Neutral and Acidic pH. ACS Macro Lett..

[33] Park D.-J., Kim S.-C., Jang J.-B., Lee B., Lee S., Ryu B., Je J.-Y., Park W.S., Jung W.-K. (2024). Multifunctional Hydrogel Dressing Based on Fish Gelatin/Oxidized Hyaluronate for Promoting Diabetic Wound Healing. J. Mater. Chem. B.

[34] Xu J., Hsu S. (2023). Self-Healing Hydrogel as an Injectable Implant: Translation in Brain Diseases. J. Biomed. Sci..

[35] Li H., Cheng F., Wei X., Yi X., Tang S., Wang Z., Zhang Y.S., He J., Huang Y. (2021). Injectable, Self-Healing, Antibacterial, and Hemostatic N,O-Carboxymethyl Chitosan/Oxidized Chondroitin Sulfate Composite Hydrogel for Wound Dressing. Mater. Sci. Eng. C.

[36] Muir V.G., Burdick J.A. (2021). Chemically Modified Biopolymers for the Formation of Biomedical Hydrogels. Chem. Rev..

[37] He L., Szopinski D., Wu Y., Luinstra G.A., Theato P. (2015). Toward Self-Healing Hydrogels Using One-Pot Thiol–Ene Click and Borax-Diol Chemistry. ACS Macro Lett..

[38] Zhao X., Wu H., Guo B., Dong R., Qiu Y., Ma P.X. (2017). Antibacterial Anti-Oxidant Electroactive Injectable Hydrogel as Self-Healing Wound Dressing with Hemostasis and Adhesiveness for Cutaneous Wound Healing. Biomaterials.

[39] Wang C., Liang C., Wang R., Yao X., Guo P., Yuan W., Liu Y., Song Y., Li Z., Xie X. (2020). The Fabrication of a Highly Efficient Self-Healing Hydrogel from Natural Biopolymers Loaded with Exosomes for the Synergistic Promotion of Severe Wound Healing. Biomater. Sci..

[40] Li Y., Wang X., Fu Y., Wei Y., Zhao L., Tao L. (2018). Self-Adapting Hydrogel to Improve the Therapeutic Effect in Wound-Healing. ACS Appl. Mater. Interfaces.

[41] Qu J., Zhao X., Liang Y., Zhang T., Ma P.X., Guo B. (2018). Antibacterial Adhesive Injectable Hydrogels with Rapid Self-Healing, Extensibility and Compressibility as Wound Dressing for Joints Skin Wound Healing. Biomaterials.

[42] Sapuła P., Bialik-Wąs K., Malarz K. (2023). Are Natural Compounds a Promising Alternative to Synthetic Cross-Linking Agents in the Preparation of Hydrogels?. Pharmaceutics.

[43] Chen H., Cheng J., Ran L., Yu K., Lu B., Lan G., Dai F., Lu F. (2018). An Injectable Self-Healing Hydrogel with Adhesive and Antibacterial Properties Effectively Promotes Wound Healing. Carbohydr. Polym..

[44] Li W., Wang B., Zhang M., Wu Z., Wei J., Jiang Y., Sheng N., Liang Q., Zhang D., Chen S. (2020). All-Natural Injectable Hydrogel with Self-Healing and Antibacterial Properties for Wound Dressing. Cellulose.

[45] Wang H., Lu B., Zhou J., Lai J., Zheng X., Guo S.-Z., Zhang L.-M. (2025). Biobased Physicochemical Reversible Dual-Cross-Linked Hydrogel: Self-Healing, Antibacterial, Antioxidant, and Hemostatic Properties for Diabetic Wound Healing. Biomacromolecules.

[46] Basu S., Pacelli S., Paul A. (2020). Self-Healing DNA-Based Injectable Hydrogels with Reversible Covalent Linkages for Controlled Drug Delivery. Acta Biomater..

[47] Lei J., Li X., Wang S., Yuan L., Ge L., Li D., Mu C. (2019). Facile Fabrication of Biocompatible Gelatin-Based Self-Healing Hydrogels. ACS Appl. Polym. Mater..

[48] Chen M., Tian J., Liu Y., Cao H., Li R., Wang J., Wu J., Zhang Q. (2019). Dynamic Covalent Constructed Self-Healing Hydrogel for Sequential Delivery of Antibacterial Agent and Growth Factor in Wound Healing. Chem. Eng. J..

[49] Li Q., Liu C., Wen J., Wu Y., Shan Y., Liao J. (2017). The Design, Mechanism and Biomedical Application of Self-Healing Hydrogels. Chin. Chem. Lett..

[50] Yang Y., Urban M.W. (2013). Self-Healing Polymeric Materials. Chem. Soc. Rev..

[51] Chen H., Cheng R., Zhao X., Zhang Y., Tam A., Yan Y., Shen H., Zhang Y.S., Qi J., Feng Y. (2019). An Injectable Self-Healing Coordinative Hydrogel with Antibacterial and Angiogenic Properties for Diabetic Skin Wound Repair. NPG Asia Mater..

[52] Morozova S.M. (2023). Recent Advances in Hydrogels via Diels–Alder Crosslinking: Design and Applications. Gels.

[53] Yu F., Cao X., Li Y., Zeng L., Zhu J., Wang G., Chen X. (2014). Diels–Alder Crosslinked HA/PEG Hydrogels with High Elasticity and Fatigue Resistance for Cell Encapsulation and Articular Cartilage Tissue Repair. Polym Chem.

[54] Ilochonwu B.C., van der Lugt S.A., Annala A., Di Marco G., Sampon T., Siepmann J., Siepmann F., Hennink W.E., Vermonden T. (2023). Thermo-Responsive Diels-Alder Stabilized Hydrogels for Ocular Drug Delivery of a Corticosteroid and an Anti-VEGF Fab Fragment. J. Control. Release.

[55] Chen Y., Wang W., Wu D., Nagao M., Hall D.G., Thundat T., Narain R. (2018). Injectable Self-Healing Zwitterionic Hydrogels Based on Dynamic Benzoxaborole–Sugar Interactions with Tunable Mechanical Properties. Biomacromolecules.

[56] Wu D., Wang W., Diaz-Dussan D., Peng Y.-Y., Chen Y., Narain R., Hall D.G. (2019). In Situ Forming, Dual-Crosslink Network, Self-Healing Hydrogel Enabled by a Bioorthogonal Nopoldiol–Benzoxaborolate Click Reaction with a Wide pH Range. Chem. Mater..

[57] Amaral A.J.R., Emamzadeh M., Pasparakis G. (2018). Transiently Malleable Multi-Healable Hydrogel Nanocomposites Based on Responsive Boronic Acid Copolymers. Polym. Chem..

[58] Liang Y., Li M., Yang Y., Qiao L., Xu H., Guo B. (2022). pH/Glucose Dual Responsive Metformin Release Hydrogel Dressings with Adhesion and Self-Healing via Dual-Dynamic Bonding for Athletic Diabetic Foot Wound Healing. ACS Nano.

[59] Chen Y., Diaz-Dussan D., Wu D., Wang W., Peng Y.-Y., Asha A.B., Hall D.G., Ishihara K., Narain R. (2018). Bioinspired Self-Healing Hydrogel Based on Benzoxaborole-Catechol Dynamic Covalent Chemistry for 3D Cell Encapsulation. ACS Macro Lett..

[60] Zhou L., Dai C., Fan L., Jiang Y., Liu C., Zhou Z., Guan P., Tian Y., Xing J., Li X. (2021). Injectable Self-Healing Natural Biopolymer-Based Hydrogel Adhesive with Thermoresponsive Reversible Adhesion for Minimally Invasive Surgery. Adv. Funct. Mater..

[61] Guan Y., Zhang Y. (2013). Boronic Acid-Containing Hydrogels: Synthesis and Their Applications. Chem. Soc. Rev..

[62] Ding X., Li G., Zhang P., Jin E., Xiao C., Chen X. (2021). Injectable Self-Healing Hydrogel Wound Dressing with Cysteine-Specific On-Demand Dissolution Property Based on Tandem Dynamic Covalent Bonds. Adv. Funct. Mater..

[63] Yang G., Zhang Z., Liu K., Ji X., Fatehi P., Chen J. (2022). A Cellulose Nanofibril-Reinforced Hydrogel with Robust Mechanical, Self-Healing, pH-Responsive and Antibacterial Characteristics for Wound Dressing Applications. J. Nanobiotechnol..

[64] Wei H., Jing H., Cheng C., Liu Y., Hao J. (2025). A Biomimetic One-Stone-Two-Birds Hydrogel with Electroconductive, Photothermally Antibacterial and Bioadhesive Properties for Skin Tissue Regeneration and Mechanosensation Restoration. Adv. Funct. Mater..

[65] Qiao L., Liang Y., Chen J., Huang Y., Alsareii S.A., Alamri A.M., Harraz F.A., Guo B. (2023). Antibacterial Conductive Self-Healing Hydrogel Wound Dressing with Dual Dynamic Bonds Promotes Infected Wound Healing. Bioact. Mater..

[66] Li M., Liang Y., He J., Zhang H., Guo B. (2020). Two-Pronged Strategy of Biomechanically Active and Biochemically Multifunctional Hydrogel Wound Dressing to Accelerate Wound Closure and Wound Healing. Chem. Mater..

[67] Ali A., Govindharaj M., Fatma B., Alshehhi K.H., Islayem D., Alsaafeen N.B., Pappa A.M., Pitsalidis C. (2025). In Situ Development of Self-Healing, Injectable, Glucose and pH-Responsive Electroconductive Composite Hydrogels. Adv. Compos. Hybrid Mater..

[68] Zhu W., Zhang J., Wei Z., Zhang B., Weng X. (2023). Advances and Progress in Self-Healing Hydrogel and Its Application in Regenerative Medicine. Materials.

[69] Wu H.-D., Yang J.-C., Tsai T., Ji D.-Y., Chang W.-J., Chen C.-C., Lee S.-Y. (2011). Development of a Chitosan–Polyglutamate Based Injectable Polyelectrolyte Complex Scaffold. Carbohydr. Polym..

[70] Phadke A., Zhang C., Arman B., Hsu C.-C., Mashelkar R.A., Lele A.K., Tauber M.J., Arya G., Varghese S. (2012). Rapid Self-Healing Hydrogels. Proc. Natl. Acad. Sci. USA.

[71] Ye X., Li X., Shen Y., Chang G., Yang J., Gu Z. (2017). Self-Healing pH-Sensitive Cytosine- and Guanosine-Modified Hyaluronic Acid Hydrogels via Hydrogen Bonding. Polymer.

[72] Cheng R., Xu M., Zhang X., Jiang J., Zhang Q., Zhao Y. (2023). Hydrogen Bonding Enables Polymer Hydrogels with pH-Induced Reversible Dynamic Responsive Behaviors. Angew. Chem. Int. Ed..

[73] Wang Y., Yang M., Zhao Z. (2023). Facile Fabrication of Self-Healing, Injectable and Antimicrobial Cationic Guar Gum Hydrogel Dressings Driven by Hydrogen Bonds. Carbohydr. Polym..

[74] Appel E.A., Tibbitt M.W., Webber M.J., Mattix B.A., Veiseh O., Langer R. (2015). Self-Assembled Hydrogels Utilizing Polymer–Nanoparticle Interactions. Nat. Commun..

[75] Owusu-Nkwantabisah S., Gillmor J.R., Switalski S.C., Slater G.L. (2017). An Autonomous Self-healing Hydrogel Based on Surfactant-free Hydrophobic Association. J. Appl. Polym. Sci..

[76] Chang X., Geng Y., Cao H., Zhou J., Tian Y., Shan G., Bao Y., Wu Z.L., Pan P. (2018). Dual-Crosslink Physical Hydrogels with High Toughness Based on Synergistic Hydrogen Bonding and Hydrophobic Interactions. Macromol. Rapid Commun..

[77] Algi M.P., Okay O. (2014). Highly Stretchable Self-Healing Poly(N,N-Dimethylacrylamide) Hydrogels. Eur. Polym. J..

[78] Gulyuz U., Okay O. (2015). Self-Healing Poly(Acrylic Acid) Hydrogels: Effect of Surfactant. Macromol. Symp..

[79] Holten-Andersen N., Harrington M.J., Birkedal H., Lee B.P., Messersmith P.B., Lee K.Y.C., Waite J.H. (2011). pH-Induced Metal-Ligand Cross-Links Inspired by Mussel Yield Self-Healing Polymer Networks with near-Covalent Elastic Moduli. Proc. Natl. Acad. Sci. USA.

[80] Liang Y., Li Z., Huang Y., Yu R., Guo B. (2021). Dual-Dynamic-Bond Cross-Linked Antibacterial Adhesive Hydrogel Sealants with On-Demand Removability for Post-Wound-Closure and Infected Wound Healing. ACS Nano.

[81] Deng K., Huang Q., Yan X., Dai Y., Zhao J., Xiong X., Wang H., Chen X., Chen P., Liu L. (2024). Facile Fabrication of a Novel, Photodetachable Salecan-Based Hydrogel Dressing with Self-Healing, Injectable, and Antibacterial Properties Based on Metal Coordination. Int. J. Biol. Macromol..

[82] Li X., Chen X., Guan L., He W., Yin W., Ye D., Gao J., Wang M., Pan G. (2024). Bioactive Metal Ion-Coordinated Dynamic Hydrogel with Antibacterial, Immunomodulatory, and Angiogenic Activities for Infected Wound Repair. ACS Appl. Mater. Interfaces.

[83] Xu X., Jerca V.V., Hoogenboom R. (2020). Self-Healing Metallo-Supramolecular Hydrogel Based on Specific Ni ^2+^ Coordination Interactions of Poly(Ethylene Glycol) with Bistriazole Pyridine Ligands in the Main Chain. Macromol. Rapid Commun..

[84] Sun Y., Gao C., Jia P., Song L., Kang J., Han M., Yu W., Nian R. (2025). Novel in Situ and Rapid Self-Gelation Recombinant Collagen-like Protein Hydrogel for Wound Regeneration: Mediated by Metal Coordination Crosslinking and Reinforced by Electro-Oxidized Tea Polyphenols. Biofabrication.

[85] Qin H., Zhang T., Li H.-N., Cong H.-P., Antonietti M., Yu S.-H. (2017). Dynamic Au-Thiolate Interaction Induced Rapid Self-Healing Nanocomposite Hydrogels with Remarkable Mechanical Behaviors. Chem.

[86] Shi L., Zhao Y., Xie Q., Fan C., Hilborn J., Dai J., Ossipov D.A. (2018). Moldable Hyaluronan Hydrogel Enabled by Dynamic Metal–Bisphosphonate Coordination Chemistry for Wound Healing. Adv. Healthc. Mater..

[87] Zeng L., Song M., Gu J., Xu Z., Xue B., Li Y., Cao Y. (2019). A Highly Stretchable, Tough, Fast Self-Healing Hydrogel Based on Peptide–Metal Ion Coordination. Biomimetics.

[88] Zhang K., Yuan W., Wei K., Yang B., Chen X., Li Z., Zhang Z., Bian L. (2019). Highly Dynamic Nanocomposite Hydrogels Self-Assembled by Metal Ion-Ligand Coordination. Small.

[89] Deng Z., Guo Y., Zhao X., Ma P.X., Guo B. (2018). Multifunctional Stimuli-Responsive Hydrogels with Self-Healing, High Conductivity, and Rapid Recovery through Host–Guest Interactions. Chem. Mater..

[90] Zhang B., He J., Shi M., Liang Y., Guo B. (2020). Injectable Self-Healing Supramolecular Hydrogels with Conductivity and Photo-Thermal Antibacterial Activity to Enhance Complete Skin Regeneration. Chem. Eng. J..

[91] Zhang Y., Xu Z., Li M., Yuan Y., Wang W., Zhang L., Wan P. (2024). Mussel-Inspired Self-Healing Adhesive MXene Hydrogel for Epidermal Electronics. Device.

[92] Khan A., Rehman W., Alanazi M.M., Khan Y., Rasheed L., Saboor A., Iqbal S. (2023). Development of Novel Multifunctional Electroactive, Self-Healing, and Tissue Adhesive Scaffold to Accelerate Cutaneous Wound Healing and Hemostatic Materials. ACS Omega.

[93] Zhao N., Yuan W. (2022). Functionally Integrated Bioglass Microspheres-Composited Double-Network Hydrogel with Good Tissue Adhesion and Electrical Conductivity for Efficient Wound Treatment and Health Detection. Compos. Part B Eng..

[94] Zhang J., Wu C., Xu Y., Chen J., Ning N., Yang Z., Guo Y., Hu X., Wang Y. (2020). Highly Stretchable and Conductive Self-Healing Hydrogels for Temperature and Strain Sensing and Chronic Wound Treatment. ACS Appl. Mater. Interfaces.

[95] Li M., Zhang Y., Lian L., Liu K., Lu M., Chen Y., Zhang L., Zhang X., Wan P. (2022). Flexible Accelerated-Wound-Healing Antibacterial MXene-Based Epidermic Sensor for Intelligent Wearable Human-Machine Interaction. Adv. Funct. Mater..

[96] Liu N., Ma H., Li M., Qin R., Li P. (2024). Electroconductive Hydrogels for Bioelectronics: Challenges and Opportunities. FlexMat.

[97] Wang J., Yang B., Jiang Z., Liu Y., Zhou L., Liu Z., Tang L. (2024). Recent Advances of Conductive Hydrogels for Flexible Electronics. Electron. Mater..

[98] Liang Y., Qiao L., Qiao B., Guo B. (2023). Conductive Hydrogels for Tissue Repair. Chem. Sci..

[99] Calderón Moreno J.M., Chelu M., Popa M. (2025). Eco-Friendly Conductive Hydrogels: Towards Green Wearable Electronics. Gels.

[100] Li Y., Tan S., Zhang X., Li Z., Cai J., Liu Y. (2025). Design Strategies and Emerging Applications of Conductive Hydrogels in Wearable Sensing. Gels.

[101] Guo X., Facchetti A. (2020). The Journey of Conducting Polymers from Discovery to Application. Nat. Mater..

[102] Sazcı O., Uğraşkan V., Hazar A.B.Y. (2023). Conductive Polymers for Medical Applications. Handbook of Polymers in Medicine.

[103] Dias D., Resina L., Ferreira F.C., Sanjuan-Alberte P., Esteves T. (2024). Synthesis Strategies and Cancer Therapy Applications of PEDOT Nanoparticles. Mater. Adv..

[104] Talikowska M., Fu X., Lisak G. (2019). Application of Conducting Polymers to Wound Care and Skin Tissue Engineering: A Review. Biosens. Bioelectron..

[105] Ansari S.P., Anis A. (2018). Conducting Polymer Hydrogels. Polymeric Gels.

[106] Sun Z., Ou Q., Dong C., Zhou J., Hu H., Li C., Huang Z. (2024). Conducting Polymer Hydrogels Based on Supramolecular Strategies for Wearable Sensors. Exploration.

[107] Lee J.-J., Ng H.Y., Lin Y.-H., Liu E.-W., Lin T.-J., Chiu H.-T., Ho X.-R., Yang H.-A., Shie M.-Y. (2022). The 3D Printed Conductive Grooved Topography Hydrogel Combined with Electrical Stimulation for Synergistically Enhancing Wound Healing of Dermal Fibroblast Cells. Biomater. Adv..

[108] Zhou X., Yu X., You T., Zhao B., Dong L., Huang C., Zhou X., Xing M., Qian W., Luo G. (2024). 3D Printing-Based Hydrogel Dressings for Wound Healing. Adv. Sci..

[109] Li Y., Peng W., Dong Y., Fan B., Qian W., Ji X., Lu X., Gan D., Liu P. (2023). Mussel-Inspired PEDOT-Incorporated Gelatin-Based Conductive Hydrogel with Flexibility and Electroactivity to Accelerate Wound Healing In Vitro. ACS Appl. Polym. Mater..

[110] Gan D., Han L., Wang M., Xing W., Xu T., Zhang H., Wang K., Fang L., Lu X. (2018). Conductive and Tough Hydrogels Based on Biopolymer Molecular Templates for Controlling in Situ Formation of Polypyrrole Nanorods. ACS Appl. Mater. Interfaces.

[111] Tang L., Xie S., Wang D., Wei Y., Ji X., Wang Y., Zhao N., Mou Z., Li B., Sun W.R. (2025). Astragalus Polysaccharide/Carboxymethyl Chitosan/Sodium Alginate Based Electroconductive Hydrogels for Diabetic Wound Healing and Muscle Function Assessment. Carbohydr. Polym..

[112] Lu Y., Wang Y., Zhang J., Hu X., Yang Z., Guo Y., Wang Y. (2019). In-Situ Doping of a Conductive Hydrogel with Low Protein Absorption and Bacterial Adhesion for Electrical Stimulation of Chronic Wounds. Acta Biomater..

[113] Guan L., Ou X., Wang Z., Li X., Feng Y., Yang X., Qu W., Yang B., Lin Q. (2023). Electrical Stimulation-Based Conductive Hydrogel for Immunoregulation, Neuroregeneration and Rapid Angiogenesis in Diabetic Wound Repair. Sci. China Mater..

[114] Lin X., Yang X., Li P., Xu Z., Zhao L., Mu C., Li D., Ge L. (2023). Antibacterial Conductive Collagen-Based Hydrogels for Accelerated Full-Thickness Wound Healing. ACS Appl. Mater. Interfaces.

[115] Xiao L., Hui F., Tian T., Yan R., Xin J., Zhao X., Jiang Y., Zhang Z., Kuang Y., Li N. (2021). A Novel Conductive Antibacterial Nanocomposite Hydrogel Dressing for Healing of Severely Infected Wounds. Front. Chem..

[116] Wu C., Shen L., Lu Y., Hu C., Liang Z., Long L., Ning N., Chen J., Guo Y., Yang Z. (2021). Intrinsic Antibacterial and Conductive Hydrogels Based on the Distinct Bactericidal Effect of Polyaniline for Infected Chronic Wound Healing. ACS Appl. Mater. Interfaces.

[117] Jayaprakash N., Elumalai K., Manickam S., Bakthavatchalam G., Tamilselvan P. (2024). Carbon Nanomaterials: Revolutionizing Biomedical Applications with Promising Potential. Nano Mater. Sci..

[118] Stocco T., Zhang T., Dimitrov E., Ghosh A., da Silva A., Melo W., Tsumura W., Silva A., Sousa G., Viana B. (2023). Carbon Nanomaterial-Based Hydrogels as Scaffolds in Tissue Engineering: A Comprehensive Review. Int. J. Nanomed..

[119] Fan Z., Liu B., Wang J., Zhang S., Lin Q., Gong P., Ma L., Yang S. (2014). A Novel Wound Dressing Based on Ag/Graphene Polymer Hydrogel: Effectively Kill Bacteria and Accelerate Wound Healing. Adv. Funct. Mater..

[120] Zmejkoski D.Z., Marković Z.M., Mitić D.D., Zdravković N.M., Kozyrovska N.O., Bugárová N., Marković B.M.T. (2022). Antibacterial Composite Hydrogels of Graphene Quantum Dots and Bacterial Cellulose Accelerate Wound Healing. J. Biomed. Mater. Res. Part B Appl. Biomater..

[121] Liang Y., Zhao X., Hu T., Han Y., Guo B. (2019). Mussel-Inspired, Antibacterial, Conductive, Antioxidant, Injectable Composite Hydrogel Wound Dressing to Promote the Regeneration of Infected Skin. J. Colloid Interface Sci..

[122] Ravanbakhsh H., Bao G., Mongeau L. (2020). Carbon Nanotubes Promote Cell Migration in Hydrogels. Sci. Rep..

[123] Wang J., He J., Zhou R., Zeng R., Guan S., Yang X., Liu Z., Liu Y., Zhu X., Liao Q. (2025). Accelerated Diabetic Wound Healing via Electrical and Oxidative Microenvironment Regulation by MXene Nanosheet-Based Hydrogel Dressings. ACS Appl. Nano Mater..

[124] Liu Z., Ma Z., Li N., Zhang J., Li M., Han L., Cheng R., Shen Z., Han D., Sang S. (2025). CS/Gel/MWCNTs Conductive Scaffolds Assisted by Electrical Stimulus for Skin Tissue Engineering. Biotechnol. Bioeng..

[125] Liu D., Bi S., Wang H., Gu J., Wang S. (2024). Polydopamine Interface-Modulated MXene-Based Conductive Antibacterial Hydrogels for on-Skin Health Monitoring and Diabetic Wound Healing. Compos. Part A Appl. Sci. Manuf..

[126] Gao C., Song S., Lv Y., Huang J., Zhang Z. (2022). Recent Development of Conductive Hydrogels for Tissue Engineering: Review and Perspective. Macromol. Biosci..

[127] Min J.H., Patel M., Koh W.-G. (2018). Incorporation of Conductive Materials into Hydrogels for Tissue Engineering Applications. Polymers.

[128] Zheng W., Yang W., Wei W., Liu Z., Tremblay P., Zhang T. (2024). An Electroconductive and Antibacterial Adhesive Nanocomposite Hydrogel for High-Performance Skin Wound Healing. Adv. Healthc. Mater..

[129] Hu X.Q., Zhu J.Z., Hao Z., Tang L., Sun J., Sun W.R., Hu J., Wang P.Y., Basmadji N.P., Pedraz J.L. (2024). Renewable Electroconductive Hydrogels for Accelerated Diabetic Wound Healing and Motion Monitoring. Biomacromolecules.

[130] Mahmoud N.N., Hikmat S., Ghith D.A., Hajeer M., Hamadneh L., Qattan D., Khalil E.A. (2019). Gold Nanoparticles Loaded into Polymeric Hydrogel for Wound Healing in Rats: Effect of Nanoparticles’ Shape and Surface Modification. Int. J. Pharm..

[131] Zheng M., Wang X., Yue O., Hou M., Zhang H., Beyer S., Blocki A.M., Wang Q., Gong G., Liu X. (2021). Skin-Inspired Gelatin-Based Flexible Bio-Electronic Hydrogel for Wound Healing Promotion and Motion Sensing. Biomaterials.

[132] Ren D., Zhang Y., Du B., Wang L., Gong M., Zhu W. (2024). An Antibacterial, Conductive Nanocomposite Hydrogel Coupled with Electrical Stimulation for Accelerated Wound Healing. Int. J. Nanomed..

[133] Ayreen Z., Khatoon U., Kirti A., Sinha A., Gupta A., Lenka S.S., Yadav A., Mohanty R., Naser S.S., Mishra R. (2024). Perilous Paradigm of Graphene Oxide and Its Derivatives in Biomedical Applications: Insight to Immunocompatibility. Biomed. Pharmacother..

[134] Brumberg V., Astrelina T., Malivanova T., Samoilov A. (2021). Modern Wound Dressings: Hydrogel Dressings. Biomedicines.

[135] Ribeiro M., Simões M., Vitorino C., Mascarenhas-Melo F. (2024). Hydrogels in Cutaneous Wound Healing: Insights into Characterization, Properties, Formulation and Therapeutic Potential. Gels.

[136] Fang Y., Han Y., Yang L., Kankala R.K., Wang S., Chen A., Fu C. (2025). Conductive Hydrogels: Intelligent Dressings for Monitoring and Healing Chronic Wounds. Regen. Biomater..

[137] Yu P., Wei L., Yang Z., Liu X., Ma H., Zhao J., Liu L., Wang L., Chen R., Cheng Y. (2024). Hydrogel Wound Dressings Accelerating Healing Process of Wounds in Movable Parts. Int. J. Mol. Sci..

[138] Chen R., Wang P., Xie J., Tang Z., Fu J., Ning Y., Zhong Q., Wang D., Lei M., Mai H. (2024). A Multifunctional Injectable, Self-Healing, and Adhesive Hydrogel-Based Wound Dressing Stimulated Diabetic Wound Healing with Combined Reactive Oxygen Species Scavenging, Hyperglycemia Reducing, and Bacteria-Killing Abilities. J. Nanobiotechnol..

[139] Alberts A., Tudorache D.-I., Niculescu A.-G., Grumezescu A.M. (2025). Advancements in Wound Dressing Materials: Highlighting Recent Progress in Hydrogels, Foams, and Antimicrobial Dressings. Gels.

[140] Liang X., Zhang M., Chong C.-M., Lin D., Chen S., Zhen Y., Ding H., Zhong H.-J. (2024). Recent Advances in the 3D Printing of Conductive Hydrogels for Sensor Applications: A Review. Polymers.

[141] Vafa Z.J., Zare E.N., Eslam M.R.F., Makvandi P. (2025). Development of Ecofriendly, Biodegradable Electrically Conductive Double-Layer Bio-Hydrogel Nanocomposite for Sustainable Medical Device Applications. Adv. Compos. Hybrid Mater..

[142] Nie L., Wei Q., Li J., Deng Y., He X., Gao X., Ma X., Liu S., Sun Y., Jiang G. (2023). Fabrication and Desired Properties of Conductive Hydrogel Dressings for Wound Healing. RSC Adv..

[143] Sui B., Liu X., Sun J. (2021). Biodistribution, Inter-/Intra-Cellular Localization and Respiratory Dysfunction Induced by Ti_3_C_2_ Nanosheets: Involvement of Surfactant Protein down-Regulation in Alveolar Epithelial Cells. J. Hazard. Mater..

[144] Sosa S., Tubaro A., Carlin M., Ponti C., Vázquez E., Prato M., Pelin M. (2023). Assessment of Skin Sensitization Properties of Few-Layer Graphene and Graphene Oxide through the Local Lymph Node Assay (OECD TG 442B). NanoImpact.

[145] Guo B., Glavas L., Albertsson A.-C. (2013). Biodegradable and Electrically Conducting Polymers for Biomedical Applications. Prog. Polym. Sci..

[146] Jadoun S., Riaz U., Budhiraja V. (2021). Biodegradable Conducting Polymeric Materials for Biomedical Applications: A Review. Med. Devices Sens..

[147] Luo R., Dai J., Zhang J., Li Z. (2021). Accelerated Skin Wound Healing by Electrical Stimulation. Adv. Healthc. Mater..

[148] Rabbani M., Rahman E., Powner M.B., Triantis I.F. (2024). Making Sense of Electrical Stimulation: A Meta-Analysis for Wound Healing. Ann. Biomed. Eng..

[149] Rizzo D., Javidi D., Werpachowski N., Frasier K., Maher B., Huh Y., Shah S., Althobaiti R.F. (2025). Advancing Wound Healing Using Cutaneous Bioelectronic Interfaces for Real-Time Monitoring and Electrical Stimulation. Dermis.

[150] Chen M., Liu H., Chen X., Kang L., Yao X., Tan L., Zhu W., Yu J., Qin X., Wu D. (2024). A Novel Multifunction of Wearable Ionic Conductive Hydrogel Sensor for Promoting Infected Wound Healing. Appl. Mater. Today.

[151] Wang L., Zhou M., Xu T., Zhang X. (2022). Multifunctional Hydrogel as Wound Dressing for Intelligent Wound Monitoring. Chem. Eng. J..

[152] Wang L., Xu T., Zhang X. (2021). Multifunctional Conductive Hydrogel-Based Flexible Wearable Sensors. TrAC Trends Anal. Chem..

[153] She Y., Liu H., Yuan H., Li Y., Liu X., Liu R., Wang M., Wang T., Wang L., Liu M. (2025). Artificial Intelligence-Assisted Conductive Hydrogel Dressings for Refractory Wounds Monitoring. Nano-Micro Lett..

[154] Finster R., Sankaran P., Bihar E. (2025). Computational and AI-Driven Design of Hydrogels for Bioelectronic Applications. Adv. Electron. Mater..

[155] Damiati L.A., Alsudir S.A., Mohammed R.Y., Majrashi M.A., Albrahim S.H., algethami A., Alghamdi F.O., Alamari H.A., Alzaydi M.M. (2025). 4D Printing in Skin Tissue Engineering: A Revolutionary Approach to Enhance Wound Healing and Combat Infections. Bioprinting.

[156] Naghib S.M., Hosseini S.N., Beigi A. (2024). 3D 4D Printing of Chitosan-Based Scaffolds for Wound Dressing Applications. Carbohydr. Polym. Technol. Appl..

[157] Lu Z., Cui J., Liu F., Liang C., Feng S., Sun Y., Gao W., Guo Y., Zhang B., Huang W. (2024). A 4D Printed Adhesive, Thermo-Contractile, and Degradable Hydrogel for Diabetic Wound Healing. Adv. Healthc. Mater..

[158] Flynn K., Mahmoud N.N., Sharifi S., Gould L.J., Mahmoudi M. (2023). Chronic Wound Healing Models. ACS Pharmacol. Transl. Sci..

